# Development of a GMMA-based candidate vaccine against invasive nontyphoidal *Salmonella* disease

**DOI:** 10.3389/fimmu.2026.1821495

**Published:** 2026-06-02

**Authors:** Francesco Citiulo, Oliver Koeberling, Omar Rossi, Emilia Cappelletti, Carlo Giannelli, Maria Grazia Aruta, Daniele De Simone, Federico Pippi, Xhenti Ferhati, Antonia De Felice, Alessandra Acquaviva, Angela Daniele, Filomena De Luca, Giulia Iannello, Claudia G. Vitali, Francesca Necchi, Laura B. Martin, Francesco Berlanda Scorza, Anna Maria Colucci, Rocío Canals

**Affiliations:** 1 GSK Vaccines Institute for Global Health S.r.l. (GVGH), Siena, Italy; 2 VectorY Therapeutics, Amsterdam, Netherlands; 3 GSK Vaccines, Siena, Italy; 4 CEPI (Coalition for Epidemic Preparedness Innovations), Washington DC, United States; 5 GSK Global Health Medicines R&D, Tres Cantos, Madrid, Spain

**Keywords:** GMMA, invasive nontyphoidal *Salmonella* (iNTS) disease, OMV, *Salmonella*, vaccine

## Abstract

**Introduction:**

Invasive nontyphoidal *Salmonella* (iNTS) disease is one of the most common causes of bacteremia in sub-Saharan Africa (sSA), mostly affecting children less than 5 years of age and with an associated 14.7% case fatality ratio. This disease is responsible for 62,018 deaths worldwide annually with more than 80% of those occurring in sSA.

**Methods:**

This work describes the pre-clinical development of a Generalized Modules for Membrane Antigens (GMMA)-based bivalent vaccine candidate against iNTS disease, known as iNTS-GMMA. *Salmonella enterica* serovars Typhimurium (*S*. Typhimurium) and Enteritidis (*S*. Enteritidis) were genetically modified to induce hyperblebbing of outer membrane vesicles, referred to as GMMA, with a penta-acylated lipid A of the lipopolysaccharide (LPS), characterized by a reduced induction of proinflammatory cytokine release from monocytes compared to GMMA carrying wild-type species of lipid A. GMMA derived from the genetically modified *Salmonella* strains were produced at scale with high yields and purity under both non-good manufacturing practice (GMP) conditions and under GMP conditions. A simple two-step filtration process was used to purify the GMMA drug substances. Subsequently, the *S*. Typhimurium GMMA and *S*. Enteritidis GMMA were individually formulated on Alhydrogel to produce the drug products. Mixing of the two components prior to injection results in the iNTS-GMMA vaccine candidate.

**Results:**

The iNTS-GMMA vaccine candidate induced in mice and rabbits high levels of IgG anti-*S*. Typhimurium and *S*. Enteritidis LPS O-antigens, the target antigens of the vaccine. Moreover, animal sera elicited strong *in vitro* bactericidal activity against *S*. Typhimurium and *S*. Enteritidis strains. Additionally, the highest envisioned human dose of iNTS-GMMA was well tolerated in a GLP repeated-dose toxicology study in rabbits.

**Conclusions:**

These data supported clinical evaluation of this urgently needed vaccine against iNTS disease in sSA.

## 1 Introduction

In sub-Saharan Africa (sSA), nontyphoidal *Salmonella* (NTS), predominantly *Salmonella enterica* serovars Typhimurium and Enteritidis (1), are a major cause of bloodstream infections known as invasive NTS (iNTS) disease (2). This febrile disease usually presents without symptoms of enterocolitis, which are frequently observed in NTS infections in high-income countries (2). Diagnosis requires blood culture and, in resource-limited healthcare system settings, most cases go undetected or are misdiagnosed making iNTS disease cases underestimated. Young children are particularly susceptible, especially those with malnutrition, malaria or HIV infection. Among African adults, iNTS is largely restricted to those with HIV infection (3). The case fatality rate of iNTS disease was estimated to be 14.7%, being higher in African children aged under 5 years old (4). The burden of this disease in sSA, together with the associated multidrug-resistance (MDR) of NTS isolates (5–10) and the lack of a licensed vaccine against iNTS disease, make vaccine development a high priority for successful public health intervention (11).

Increase of antibodies against NTS surface polysaccharides (O-antigen, OAg) with age in African children was observed to be associated with a decrease in cases of iNTS disease (9). These antibodies elicited killing by complement and through opsonization by phagocytes (12). Previous studies in mice conducted using laboratory strains of NTS indicated that antibodies against OAg can elicit protective immunity (13). Additionally, immunization with *S.* Typhimurium and *S.* Enteritidis OAg-conjugate vaccines conferred protection against subsequent challenge in mice (14). We previously demonstrated that immunizing mice and rabbits with heat-inactivated wild-type *S.* Typhimurium strain D23580, isolated from the blood of a Malawian child, induced a strong bactericidal response mainly against the OAg unlike rough mutants of D23580 lacking the OAg (15). Bactericidal ability of anti-OAg antibodies was confirmed following purification of these antibodies (15). All these findings supported the development of a vaccine with the OAg of *S.* Typhimurium and *S.* Enteritidis as active ingredients.

To develop a vaccine against iNTS disease, we used Generalized Modules for Membrane Antigens (GMMA) as a delivery system for *S.* Typhimurium and *S.* Enteritidis OAg. Ease of manufacture and high immunogenicity make GMMA a technology suited for vaccines designated to low- and middle-income countries (LMIC). GMMA are outer membrane vesicles shed from bacteria genetically modified to induce hyperblebbing and lower the risk of reactogenicity (16), through a modified lipid A to decrease stimulation of pro-inflammatory cytokines (17). The GMMA technology allows presentation of the OAg to the immune system in its natural conformation. The vaccines based on this technology have shown a favorable safety and tolerability profile in Phase I and II human clinical trials (18–21). The iNTS-GMMA vaccine candidate targeting serogroups O:4 (*S.* Typhimurium) and O:9 (*S.* Enteritidis) could have the potential of covering 93.8% of all *Salmonella* isolates classified to the serogroup level assuming cross-protection within the same serogroup (22). We previously reported that GMMA from *S*. Typhimurium (STmGMMA) and *S*. Enteritidis (SEnGMMA) are able to induce a strong immune response and protect against infection with African *S.* Typhimurium and *S.* Enteritidis isolates in mice (23). STmGMMA formulated on Alhydrogel have been reported to induce long-lasting humoral and cellular immune responses in mice against *S*. Typhimurium (24). Additionally, we have shown that serum antibodies induced by a bivalent formulation of STmGMMA and SEnGMMA were able to kill a broad panel of *Salmonella in vitro* in a complement-mediated fashion. Those strains included isolates causing invasive disease in Africa and Southeast Asia, global representatives causing gastroenteritis and other *S*. *enterica* serovars in addition to *S.* Typhimurium and *S.* Enteritidis (25).

The iNTS-GMMA vaccine candidate progressed into clinical development and the safety and immunogenicity was evaluated in a first-in-human, single center, randomized within cohort, placebo-controlled, dose escalation trial. No Serious Adverse Events (SAEs) or Suspected Unexpected Serious Adverse Reactions (SUSARs) were reported. The most common Adverse Events (AE) were injection site reactions (26). Immunologically, an increase from baseline in serovar-specific OAg IgG levels peaked at day 28 following full dose and SBA peaked at day 28 following first vaccination (26). Currently, an age de-escalation and dose escalation approach in African population, starting with adults (18–50 years of age), then on children (24–59 months of age) and finally to infants (9 months and 6 weeks of age) is ongoing to evaluate the safety, reactogenicity, and immune response of the vaccine in these population and ages (27).

In this study, we describe the data package that was generated to support clinical trials of the iNTS-GMMA vaccine candidate (18, 26, 28), including the feasibility of the industrial production of GMMA from genetically modified *S*. Typhimurium and *S*. Enteritidis strains, the formulation of the two vaccine components (STmGMMA/Alhydrogel and SEnGMMA/Alhydrogel) and the pre-clinical data of the iNTS-GMMA candidate vaccine.

## 2 Materials and methods

### 2.1 Generation of *S.* Typhimurium and *S.* Enteritidis GMMA vaccine production strains

*Salmonella* Typhimurium wild-type strain 2192 was provided by the *Salmonella* Genetic Stock Center (SGSC) at the University of Calgary, Canada, which belongs to the global *Salmonella* reference collection A (SARA 12). This strain was isolated from a horse in Louisiana, USA (29, 30). *Salmonella* Enteritidis wild-type strain 618 was provided by Quotient Bioresearch Limited, UK. This strain of animal origin was isolated by the European Antimicrobial Susceptibility Surveillance in Animals (EASSA). The *S*. Enteritidis strain belonged to CEESA EASSA collections II and III A (30, 31). Agglutination, as per manufacturer procedure, was carried out using the Bio-Rad monovalent antiserum to *Salmonella* somatic antigen group O:4,5 for *S*. Typhimurium and the O:9 for *S*. Enteritidis. The agglutination was performed on the wild-type isolates and at each step of the generation of the vaccine production strains. To generate the vaccine production *S.* Typhimurium NVGH2363 Δ*tolR*::*aph* Δ*msbB::tetRA* Δ*pagP*::*cat* (from strain 2192) and *S.* Enteritidis NVGH2157 Δ*tolR*::*aph* Δ*msbB::tetRA* Δ*pagP*::*cat* (from strain 618), the *tolR* gene was replaced with the kanamycin resistance gene *aph*, the *msbB* gene was replaced with the tetracycline resistance gene *tetRA* and the *pagP* gene was replaced with the chloramphenicol resistance gene *cat* using the same strategy and primers previously described (17). Recombinant mutants for *S.* Typhimurium and *S.* Enteritidis were selected and the structure of the genetically modified lipid A was characterized using the same previously described methodology (16, 17). The vaccine production strains were named respectively NVGH2363 (*S.* Typhimurium) and NVGH2157 (*S.* Enteritidis). Master Cell Banks (MCB) of *S*. Typhimurium and *S*. Enteritidis were generated from the vaccine production strains under good manufacturing practices (GMP) conditions by a contract manufacturing organization (CMO).

### 2.2 GMMA production

Fermentation and GMMA purification methods were adapted from those previously published (16, 32) to produce GMMA from *S*. Typhimurium (STmGMMA) and *S*. Enteritidis (SEnGMMA) from the respective MCBs. For the STmGMMA and SEnGMMA drug substances production lots, a starter flask with around 100 mL of Defined Medium (DM), similar to *Shigella sonnei* DM (16), was inoculated with a MCB vial of the respective bacterial strains (differences between the DM used for *S*. Enteritidis and *S*. Typhimurium fermentations and *Shigella sonnei* DM are shown in **Supplementary Table 1**). The starter flasks were used to inoculate the inoculum flasks starting from 0.2 to 0.6 OD_600_ with agitation (200 rpm) at 30 °C until the OD_600_ reached 6.0 ± 3.0. Cultures were subsequently used to inoculate either a 30 L of DM in a Sartorius, Biostat D75 Bioreactor (GSK Vaccines Institute for Global Health, GVGH, consistency runs), or 25 L of DM in a LP35 Bioengineering Bioreactor (CMO GMP batches), at an inoculum size of 2% and grown at 30 °C to a final OD_600_ of 40 ± 15. The fermentation conditions were controlled to pH 6.7 kept by addition of 25–28% NH_4_OH, 30 °C, dissolved oxygen kept at 30%, airflow 15 to 30 standard liters per minute (SLPM), stir speed 200–800 rpm (cascade mode), until the final OD was reached. GMMA were purified using similar methods previously reported for *S. sonnei* and *Shigella flexneri* GMMA (16, 32) by either GVGH (non-GMP) or under GMP conditions by a CMO using defined batch production records. Briefly, GMMA released into the fermentation broth were purified using two consecutive Tangential Flow Filtration (TFF) steps. First, a microfiltration was performed to separate the culture supernatant containing the GMMA from bacteria, and second, an ultrafiltration was subsequently performed in which the GMMA were separated from soluble proteins and nucleic acids. The purified GMMA were stored at -80 °C. GVGH consistency runs produced the non-GMP GMMA reference standards and the CMO produced the GMP GMMA drug substances that were later used for the production of the toxicology and clinical lots. For drug substances lot release, GMMA characterization included total OAg content (HPAEC-PAD), total protein content (micro-BCA) and OAg/total protein (ratio), lipid A amount (HPLC-RP MS), particle size (dynamic light scattering). The percentage of soluble proteins was determined by protein quantification in the supernatant after GMMA ultracentrifugation. The OAg was extracted after treatment with acetic acid and characterized for O-acetyl content by NMR or Hestrin colorimetric method. OAg molecular size distribution was measured by HPLC-SEC/semicarbazide, and lipid A structure was confirmed by MALDI-TOF applying previously described procedures (32, 33).

### 2.3 Formulation of the iNTS-GMMA vaccine components

#### 2.3.1 STmGMMA and SEnGMMA formulation on Alhydrogel

STmGMMA and SEnGMMA drug product formulations were prepared at 1 L scale starting from a sterile filtered (using a Sartobran P150) suspension of the respective GMMA in 154 mM NaCl. This solution was added to aluminum hydroxide (Alhydrogel, 2% Brenntag, wet gel suspension) in water to reach the final OAg concentration of 80 µg/mL and stirred (90 rpm) for 2 h at room temperature. NaCl and phosphate buffers were added to achieve the desired osmolality and pH and then aliquoted at 0.7 mL in 3 mL glass vials and stored at 2-8 °C. The final formulation of each vaccine component (STmGMMA/Alhydrogel and SEnGMMA/Alhydrogel) contained 80 µg/mL OAg and 0.7 mg/mL Alhydrogel suspended in sodium phosphate-buffered saline. The formulations were tested for identity, OAg content, total protein content, aluminum content, extractable volume, non-adsorbed protein, visual appearance, pH, osmolality, sterility, immunogenicity and were placed on stability studies. GVGH consistency runs produced the reference standards and the CMO produced the non-GMP toxicology lots and the GMP clinical lots for Phase I. For the Phase I study, a Placebo was also prepared under GMP conditions using Alhydrogel, 0.7 mg/mL, and was named iNTS Placebo/Alhydrogel diluent. The Placebo lot was tested for identity, aluminum content, extractable volume, visual appearance, pH and osmolality. The STmGMMA/Alhydrogel, SEnGMMA/Alhydrogel and Placebo lots met the defined specifications.

#### 2.3.2 The bivalent iNTS-GMMA vaccine

The STmGMMA/Alhydrogel and SEnGMMA/Alhydrogel lots are combined prior to injection to generate the bivalent iNTS-GMMA vaccine candidate. A mixing protocol was developed to define robust and reproducible methods which ensured dose, uniformity, stability and absence of contamination. This protocol was used for *in vivo* vaccine evaluation of pyrogenicity and toxicity in rabbits and was used for the Phase I clinical trial (26). Briefly, 0.5 mL each of STmGMMA/Alhydrogel (40 µg OAg) and SEnGMMA/Alhydrogel (40 µg OAg) were removed from the original vial and added to a third sterile sealed vial and gently mixed. The bivalent iNTS-GMMA vaccine candidate thus contained 20 µg OAg of *S*. Typhimurium GMMA, 20 µg OAg of *S*. Enteritidis, GMMA and 0.35 mg Alhydrogel in 0.5 mL delivery volume. The iNTS-GMMA vaccine candidate was characterized after mixture of the two vaccine components and in a short-term stability study for the following quality attributes: OAg content, total protein content, non-adsorbed protein, visual appearance, pH, and osmolality.

### 2.4 Physico-chemical analytical methods

The *S.* Typhimurium and *S.* Enteritidis OAg active ingredients were quantified by High-performance anion-exchange chromatography/pulsed amperometric detection (HPAEC-PAD) analyses performed directly on Alhydrogel formulation as previously described (32, 33). The OAg component identity and quantity were evaluated in the formulation by Formulated Alhydrogel competitive ELISA (FAcE) (34) or for identity by Western Blot. The degree of O-acetylation was measured using the Hestrin colorimetric method (35), and the rhamnose content was measured using the Dische colorimetric assay for methylpentoses (36). Total protein content was estimated by micro-BCA either on the GMMA or on the drug product supernatant collected after 0.2 µm centrifugal filtration by nanosep of the sample together with HPLC-RP/MS to quantify unabsorbed protein and lipid A GMMA contents, respectively (33). Light scattering was used for particle size estimation (37). Monocyte activation test (MAT) on the formulated GMMA was performed following Ph. Eur. Chapter 2.6.30 Method 2 similarly to the method used for the characterization of the alt-Sonflex1-2–3 vaccine, a multi-component *Shigella* GMMA-based vaccine containing GMMA from *S. sonnei* and *S. flexneri* 1b, 2a and 3a adsorbed on Alhydrogel (32) (38).

Data to demonstrate consistency of the process are presented for lots produced at GVGH (reference standards), lots produced under non-GMP conditions at a CMO (toxicology lots), and lots produced under GMP conditions at a second CMOs (clinical lots used in a Phase I study).

### 2.5 Preclinical *in vivo* immunogenicity and toxicology

#### 2.5.1 Immunogenicity in mice

Eight CD1 mice (female, 4 to 6 weeks old) per group received intraperitoneal injections of different OAg doses of STmGMMA/Alhydrogel, SEnGMMA/Alhydrogel or the iNTS-GMMA vaccine (0.16 µg and 2.5 µg in OAg of STm and SEn GMMA administered either as single components or as part of the iNTS-GMMA vaccine, i.e. 0.32 µg of iNTS-GMMA consisting of 0.16 µg STmGMMA and 0.16 µg STmGMMA; and 5 µg of iNTS-GMMA consisting of 2.5 µg STmGMMA and 2.5 µg STmGMMA) on days 0 and 28 in a volume of 0.5 mL. Control mice received 0.5 mL of placebo. Blood samples were collected on days 0, 21, 28 (prior to vaccine injection), and 35 or 42 depending on the studies.

In immunogenicity studies in mice, the response elicited by a range of doses of STmGMMA/Alhydrogel or SEnGMMA/Alhydrogel, administered as single components, was compared to the immunogenicity of a formulation in which the two components were mixed prior to injection (the iNTS-GMMA vaccine candidate), containing the same amounts of *S*. Typhimurium and *S*. Enteritidis GMMA. Enzyme-linked immunosorbent assay (ELISA) analyses to determine the levels of anti-*S*. Typhimurium or anti-*S*. Enteritidis OAg IgG antibodies were performed. The OAg used as coating was purified from *S*. Typhimurium 2192 Δ*tolR*::*aph* Δ*msbB::tetRA* Δ*pagP*::*cat* (NVGH2363) or from *S*. Enteritidis 618 wild-type (NVGH505) and used at 5 μg/mL and 15 μg/mL, respectively. The secondary detection antibody used was the anti-mouse IgG (γ-chain specific)-alkaline phosphatase antibody produced in goat (Sigma A3438) with a 1:4000 dilution factor. The ELISA analyses were performed as previously described (23, 30) and with the same protocol used for analysis of human sera from the vaccine clinical trials (39). Serum bactericidal assay (SBA) to evaluate the functional activity of the antibodies against *S*. Typhimurium strain D23580 and *S*. Enteritidis strain CMCC4314 was performed as previously described (25, 40). *S*. Typhimurium D23580 was isolated from a child in Malawi with bloodstream infection (41) and *S*. Enteritidis CMCC4314 is a laboratory isolate (42).

Immunogenicity studies in mice were also used as a measure of vaccine component potency as part of the stability assessment over time. Groups of mice were immunized with four different OAg doses of STmGMMA/Alhydrogel or SEnGMMA/Alhydrogel and with their respective freshly formulated GMMA potency standard. The calculated relative potency of vaccine and potency standard was determined by the estimated difference in the Y-intercept divided by estimated slope as previously described (16).

#### 2.5.2 Immunogenicity in rabbits

Twelve New Zealand White rabbits (female, >1.5 kg) were divided into 4 groups of 3 rabbits. One group was immunized intramuscularly with the highest envisioned human dose of the iNTS-GMMA vaccine prepared by mixing equal volumes of the individual components STmGMMA/Alhydrogel and SEnGMMA/Alhydrogel prior to injection (i.e. 20 μg in OAg content from STmGMMA/Alhydrogel mixed with 20 μg in OAg content from SEnGMMA/Alhydrogel, in a final volume of 0.5 mL). The other groups received single formulations of STmGMMA/Alhydrogel or SEnGMMA/Alhydrogel or placebo (Alhydrogel diluent). The rabbits were immunized on days 0 and 28, and ELISA evaluations were performed on individual sera obtained on days 21 and/or 28 (prior to vaccine injection) and 42 to determine the *S.* Typhimurium and *S.* Enteritidis anti-OAg IgG. SBA titers (IC50) against *S.* Typhimurium and *S.* Enteritidis of sera obtained on day 42 were also evaluated. Additionally, immunogenicity was assessed as part of the non-GLP immunogenicity phase of the toxicology study of the iNTS-GMMA vaccine, measuring the anti-*S.* Typhimurium and anti-*S.* Enteritidis OAg serum IgG levels of rabbits immunized with the iNTS-GMMA vaccine, compared with rabbits administered with saline, on days 15, 29, 43 and 71.

ELISA analyses to determine the levels of anti-*S*. Typhimurium or anti-*S*. Enteritidis OAg IgG antibodies were performed similarly to mice ELISA but using anti-rabbit IgG (whole molecule)–alkaline phosphatase antibody produced in goat (Sigma A3687) with a dilution factor of 1:10000 (23, 30, 39). Serum bactericidal assay (SBA) to evaluate the functional activity of the raised antibodies in rabbits against *S*. Typhimurium strain D23580 and *S*. Enteritidis strain CMCC4314 was performed as previously described (25, 40).

#### 2.5.3 Pyrogenicity in rabbits

For the assessment of GMMA-induced fever response in rabbits, a modification of the European Pharmacopeia pyrogenicity test method (Ph. Eur. 2.6.8 pyrogens (43)) was used. This modification allows intramuscular administration of the full human dose, as previously described (16). Briefly, rabbits divided into groups of three were placed in retaining boxes and body temperatures were recorded using a rectal probe. The STmGMMA/Alhydrogel and SEnGMMA/Alhydrogel vaccine components alone or when combined prior to injection to form the iNTS-GMMA vaccine were administered intramuscularly in 0.5 mL. Sterile physiological saline was injected into the three rabbits of the control group. As a comparator, the *S. sonnei* GMMA-based vaccine 1790GAHB (5.9 µg OAg/100 µg protein) was used (16).

Temperatures were recorded continuously by an automated system from 90 min before injection until 3 h after administration to determine the initial temperature and a possible temperature rise after administration. Temperature was recorded manually at 3.5, 4, 4.5 and 5 h. The maximum temperature rise for each rabbit was determined as the difference between the highest temperature measured during the 4 h period after administration and the initial temperature. For the test to be considered valid, the mean of the maximum temperature rise of three controls had to be ≤0.3 °C. The saline group served primarily as the negative control and ensured that any temperature rise observed in the rabbits was due to the test substance, not to the vehicle or to the handling process.

#### 2.5.4 Toxicity study in rabbits

A repeat dose toxicity study was conducted in New Zealand White rabbits (male and female, >1.5 kg) in compliance with Good Laboratory Practice (GLP) with iNTS-GMMA candidate, to evaluate the local tolerance and the potential systemic toxic effects including the persistence, delayed onset or reversibility of any effects over a 4-week treatment-free period after the last injection. Rabbits were assigned to two groups: one received the highest anticipated human dose of the iNTS-GMMA vaccine candidate (i.e. 20 μg in OAg content from STmGMMA/Alhydrogel mixed with 20 μg in OAg content from SEnGMMA/Alhydrogel, in a final volume of 0.5 mL, prepared by mixing the two vaccine components prior to administration as described in section 2.3.2) and a control group received 0.9% saline. The vaccine was administered intramuscularly four times, two-weeks apart (days 1, 15, 29 and 43), followed by a four-week recovery period. Half of the animals in the two groups were sacrificed on day 46 (main necropsy) and the other half at day 71 (recovery necropsy). Over the course of the study, all animals were observed for morbidity/mortality, clinical signs, injection site reactions (Draize scoring), ophthalmology, body weight and food consumption. Clinical pathology evaluations, macroscopic observations at necropsies, organs weight assessments, and histopathology (complete WHO 2005 tissue list (44)) were also performed. Body temperatures were recorded after the first immunization. A non-GLP immunogenicity phase was also included consisting of ELISA and SBA evaluations on sera collected from animals. As Alhydrogel is a well−characterized and widely used adjuvant with an extensive history of clinical use, and its safety profile is well established, in accordance with WHO guidance, the evaluation focused on the toxicity of the final adjuvanted vaccine formulation, rather than its individual components. This approach is consistent with current regulatory practice for vaccines formulated with established aluminum−based adjuvants. In addition, in alignment with the 3Rs principles (Replacement, Reduction, and Refinement), the inclusion of a saline control group was considered the most appropriate negative control, and an additional adjuvant−only group was not deemed scientifically necessary.

### 2.6 Statistical analysis

For the non-GLP studies conducted in mice and rabbits, the Mann–Whitney two-tailed test was used to compare the immune response elicited by the different formulations at the same time point. The Spearman rank test was applied to verify dose response. For the toxicology study in rabbits, the Wilcoxon matched-pairs signed-rank test was conducted to assess the responses of the rabbits prior to and after the administrations of the iNTS-GMMA vaccine candidate. Statistical analyses were performed using GraphPad Prism 8.

### 2.7 Ethics statement

All animal studies were ethically reviewed by the local Ethical Bodies and carried out in accordance with European legislation (EU DIR 63/2010) and national laws, and the GSK Policies on the Care, Welfare and Treatment of Animals.

## 3 Results

### 3.1 Production and characterization of GMMA from *S.* Typhimurium and *S*. Enteritidis vaccine strains

Cell lines were obtained from *S.* Typhimurium 2192 and *S*. Enteritidis 618 strains with knock-out mutations in the *tolR*, *msbB* and *pagP* genes and Master Cell Banks (MCBs) were produced from these cell lines under good manufacturing practices (GMP) conditions. These MCBs were used to produce GMMA from *S.* Typhimurium (STmGMMA) and *S*. Enteritidis (SEnGMMA). The STmGMMA and SEnGMMA drug substances are suspensions of outer membrane vesicles, which have been purified from the fermentation broth of the respective genetically engineered strains. For both *S.* Typhimurium and *S*. Enteritidis, the fermentation processes to obtain GMMA were optimized at 30 L to reach an OD_600_ of approximately 40 ± 15, within 16 ± 4 h from the inoculation of the bioreactor, when a dissolved pO_2_ spike occurs and the pH starts increasing. At the end of the fermentation processes, bacterial cultures were harvested by microfiltration. Subsequently, GMMA were purified by ultrafiltration. At least two 30 L consistency runs under non-GMP conditions were performed at GVGH to generate STmGMMA and SEnGMMA. The manufacturing technology and analytics were transferred to an external Contract Manufacturing Organization (CMO) for production under GMP-compliant conditions. The GMP STmGMMA and SEnGMMA batches were produced at 25 L scale. Non-GMP and GMP fermentations of *S*. Typhimurium and *S*. Enteritidis reached comparable OD values and gave a similar yield of GMMA with similar characteristics in the two fermenter systems.

Dimensional analysis by DLS showed that particle size diameter (Z-average) of the *S.* Typhimurium and *S*. Enteritidis GMMA ranged from 80 to 140 nm and from 70 to 120 nm, respectively. The average size of the OAg extracted from GMMA was assessed by size exclusion chromatography using refractive index (**Supplementary Figure 1**). For both STmGMMA and SEnGMMA, two OAg peaks were observed, a medium molecular weight peak ranging from 25 to 40 kDa and a low molecular weight peak ranging from 1 to 3 KDa. The O-acetylation of the STmGMMA OAg, quantified using the Hestrin/Dische colorimetric assay, was in line with the O-acetylation level of the wild-type *S*. Typhimurium strain. The structure of the lipid A present in STmGMMA and SEnGMMA, analysed by mass spectroscopy using MALDI-TOF, showed a predominant peak at 1585 m/z demonstrating the loss of a myristoyl fatty acid chain with respect to the wild-type species (**Supplementary Figure 2A**). In the *in vitro* MAT using human peripheral blood mononuclear cells (PBMC), the STmGMMA and SEnGMMA drug substances produced under GMP conditions from the MCBs of the vaccine production strains with genetically modified lipid A had approximately 100-fold lower ability to stimulate release of the proinflammatory cytokine Interleukin (IL)-6 than the STmGMMA or SEnGMMA from the corresponding strains with a wild-type lipid A. These MAT results obtained using GMP GMMA were similar to data reported with research strains (17) (**Supplementary Figure 2B**). The STmGMMA and SEnGMMA specifically reacted in dot blot assays with the serotype specific monovalent sera (**Supplementary Figure 3**).

Comparability of the GMMA lots produced under non-GMP conditions with the lots produced under GMP conditions was established. Selected analyses of quality attributes that were part of the release panel of the GMMA reference standards produced under non-GMP conditions compared to GMMA produced under GMP conditions are shown in **Table 1A**. The non-GMP reference standards and the GMP STmGMMA and SEnGMMA drug substances were placed on real time and accelerated stability plans for biochemical assessment. In addition, GMMA stored at -80 °C were subjected to four freeze–thaw cycles, each consisting of thawing to 25 °C followed by refreezing to -80 °C. Both STmGMMA and SEnGMMA did not show significant changes either over time or after freeze-thaw cycles, indicating that they were stable and did not aggregate under these conditions.

**Table 1A:** Summary analysis of quality attributes from release panel for STmGMMA and SEnGMMA reference lots compared to GMP lot.

Test (method, units)	STmGMMA		SEnGMMA	
	30 L pilot run (NVGH2767)	25 L GMP run (16L3160)	30 L pilot run (NVGH2848)	25 L GMP run (18C8294)
Total protein (microBCA, µg/mL)	3043	3189.2	1364.4	1810.9
Soluble protein (microBCA, µg/mL)	129	127.7	73.3	51.5
Soluble protein/total protein (calculation, %)	4	4	5.4	3
OAg quantification (HPAEC-PAD, µg/mL)	2864	2288	3063.6	4387
OAg/Total protein (calculation, %)	0.94	0.7	2.24	2.4
OAg molecular size distribution (HPLC-SEC semicharbazide, peak kDa)	Peak 1: 34.6 Peak 2: 1.9 Molar ratio Peak 1:Peak 2 1:8.8	Peak 1: 34.0 Peak 2: 2.0 Molar ratio Peak 1:Peak 2 1:12.0	Peak 1: 30 Peak 2: 2 Molar ratio Peak 1:Peak 2 1:5.0	Peak 1: 30 Peak 2: 2 Molar ratio Peak 1:Peak 2 1:4.0
OAg O-acetylation (Hestrin Dische)	0.87	2.1	n/a	n/a
Lipid A quantification (HPLC-RP QQQ, nmol/mL)	1156.0	515.7	676.9	1411.0
Particle size determination (Dynamic light scattering, Z-average nm)	108	119	46	92

**Table 1B:** Summary analysis of quality attributes from release/surveillance panel for STmGMMA/Alhydrogel and SEnGMMA/Alhydrogel toxicology and GMP batches.

Test (Method, units)	STmGMMA/Alhydrogel				SEnGMMA/Alhydrogel			
	TOX R-0007-02	GMP CMO 1 ST-20-001	GMP CMO 2 BJ0122	GMP CMO 2 BJ0222	TOX R-0007-01	GMP CMO 1 SE-20-002	GMP CMO 2 BH0122	GMP CMO 2 BH0222
	Results				Results			
O-Antigen (OAg) identification of STmGMMA/SEnGMMA Drug Product (Western blot; positive)	Conform	Conform	Conform	Conform	Conform	Conform	Conform	Conform
Total STm/SEn OAg quantification in final DP (HPAEC-PAD; µg/mL)	–	59	70	70	–	74	61	67
STm/SEn OAg in final DP (FAcE; µg/mL)	102	61.0	82.8	85.7	82.0	57.8	79.5	65.9
Total protein not adsorbed on Alhydrogel (µBCA on DP supernatant μg/mL - % for Tox)	< 10%	5.5	8.5	13.2	<10%	3.6	2.6	3.1
GMMA not adsorbed on Alhydrogel (lipid A) (HPLC-RP/MS; nmol/mL)	–	0.41	1.5	3.8	–	0.8	0.6	0.6
Total protein (µBCA on final DP; µg/mL)	–	84	85.9	86.2	–	31	30.4	30.9
Particle size D [4,3] and D(90) (Light Scattering; µm)	D [4, 3] 9.1	D [4,3]8.5 D(90) 23.9	D [4,3]10.2 D(90) 20.5	D[4,3]10.5 D(90) 26.6	D[4; 3] 7.6	D[4; 3] 8.9 D(90) 25.9	D[4; 3] 8.3 D(90) 13.2	D[4,3] 6.1 D(90)10.1
Cytokine release (MAT, RPU)	–	1.55^$^ RPU/mL (GCV 35%)	1.54^$^ RPU/mL (GCV 39%)	1.72^$^ RPU/mL (GCV 35%)	–	1.55^$^ RPU/mL (GCV 35%)	1.54^$^ RPU/mL (GCV 39%)	1.72^$^ RPU/mL (GCV 35%)

**^$^** MAT test performed as bivalent final formulation iNTS-GMMA.

### 3.2 Production and characterization of the STmGMMA/Alhydrogel and SEnGMMA/Alhydrogel vaccine components

Formulation procedures for STmGMMA and SEnGMMA based on the adsorption of the GMMA to Alhydrogel to produce the STmGMMA/Alhydrogel and SEnGMMA/Alhydrogel drug products, respectively, were developed at small scale and then scaled up. A 1 L scale was used to produce the reference standards at GVGH, the non-GMP toxicology lots and the GMP Phase I study lots at a CMO. The Alhydrogel formulations were characterized by a panel of tests (**Table 1B**) obtaining consistent results for GVGH lots and non-GMP formulations used for toxicology study.

An analytical panel was developed to characterize both the STmGMMA/Alhydrogel and SEnGMMA/Alhydrogel formulations. HPAEC-PAD and/or FAcE assays were used to quantify OAg. Micro-BCA and HPLC-RP/MS analyses were used to verify the presence of GMMA not adsorbed on Alhydrogel by quantifying total protein and lipid A content respectively. These measurements were confirmed to be at low levels in all preparations (≤10% total protein and total lipid A). **Table 1B** reports the characterization of the non-GMP toxicology lots and GMP clinical trial material (produced by two different CMOs) confirming the robustness of the process and comparability of results. Quality of the toxicology batches and the clinical trial batches was controlled, where applicable, by tests based on European Pharmacopoeia (Ph. Eur.), United States Pharmacopeia (USP) and World Health Organization (WHO) monographs as well as International Conference on Harmonization (ICH), European Medicines Agency (EMEA) and U.S. Food and Drug Administration (FDA) guidelines. Results from testing the sterility, the container closure integrity of the different lots and their appearance, the aluminum quantity and uniformity, the total volume and extractable volume confirmed that they met the specifications. The pH of the different lots ranged from 6.3 to 6.5 and the osmolality from 319 to 330 mOsm/kg.

STmGMMA/Alhydrogel and SEnGMMA/Alhydrogel non-GMP toxicology lots and GMP lots produced at a CMO were assessed up to 42 months at real time temperature (2-8 °C) and the stability results conformed to established physico-chemical parameters over the testing period. As shown in **Supplementary Table 2** for the non-GMP toxicology lots, the STmGMMA/Alhydrogel GMP lot ST-20–001 and the SEnGMMA/Alhydrogel GMP lot SE-20-002, by performing linear regression analysis of stability data, only showed minor trends over time for some of the quality attributes that did not affect product stability and remained within the range of the acceptance criteria when established. The STmGMMA/Alhydrogel and SEnGMMA/Alhydrogel toxicology lots also underwent accelerated stability assessment by storage at 25 °C and 37 °C for 56 days, **Supplementary Table 3**. The dataset over the course of the study showed no significant trend at 25 °C (with the exception of particle size [D90] for SEnGMMA/Alhydrogel for which an increasing trend was identified) and 37 °C for *S.* Typhimurium and *S.* Enteritidis OAg quantified by FAcE, total protein not adsorbed to Alhydrogel, osmolality, particle size D [3, 4] and appearance. In contrast, when *S.* Typhimurium and *S.* Enteritidis GMMA were incubated at 50 °C or higher temperatures, a significant decreasing trend was observed for the total OAg and the OAg O-acetylation dramatically decreased after 4 weeks at 50 °C in *S.* Typhimurium (37). Based on these results, the STmGMMA/Alhydrogel and SEnGMMA/Alhydrogel appeared to be stable for only short periods of time at 50 °C and, therefore, it was considered that exposure to temperatures above 25 °C for any longer than 3 days should be avoided to preserve the integrity of the drug products. Stability data in real time (2–8 °C up to 42 months) and accelerated (25 and 37 °C for up to 56 days) conditions collected so far support a shelf-life assignment of 42 months at 2–8 °C storage conditions.

### 3.3 The iNTS-GMMA vaccine candidate

The investigational iNTS-GMMA vaccine is a white suspension composed of *S.* Typhimurium and *S*. Enteritidis GMMA drug substances adsorbed on 0.7 mg/mL aluminum hydroxide (Alhydrogel 2%, wet gel suspension) suspended in sodium phosphate buffered saline. The vaccine is supplied in 2 single dose vials containing either STmGMMA/Alhydrogel or SEnGMMA/Alhydrogel (80 µg OAg/mL) and mixed shortly before administration. After the mixing procedure, 0.5 mL of the iNTS-GMMA vaccine candidate, containing STmGMMA and SEnGMMA with 20 µg OAg each, is administered intramuscularly as full dose. A mixing prior to injection protocol was developed at GVGH to consistently produce the iNTS-GMMA vaccine after mixing the STmGMMA/Alhydrogel and SEnGMMA/Alhydrogel demonstrating flexibility and consistency of the formulation approach.

### 3.4 Immunogenicity and potency of the STmGMMA/Alhydrogel, SEnGMMA/Alhydrogel vaccine components or when combined to generate the iNTS-GMMA vaccine candidate in mice

**Figure 1:**
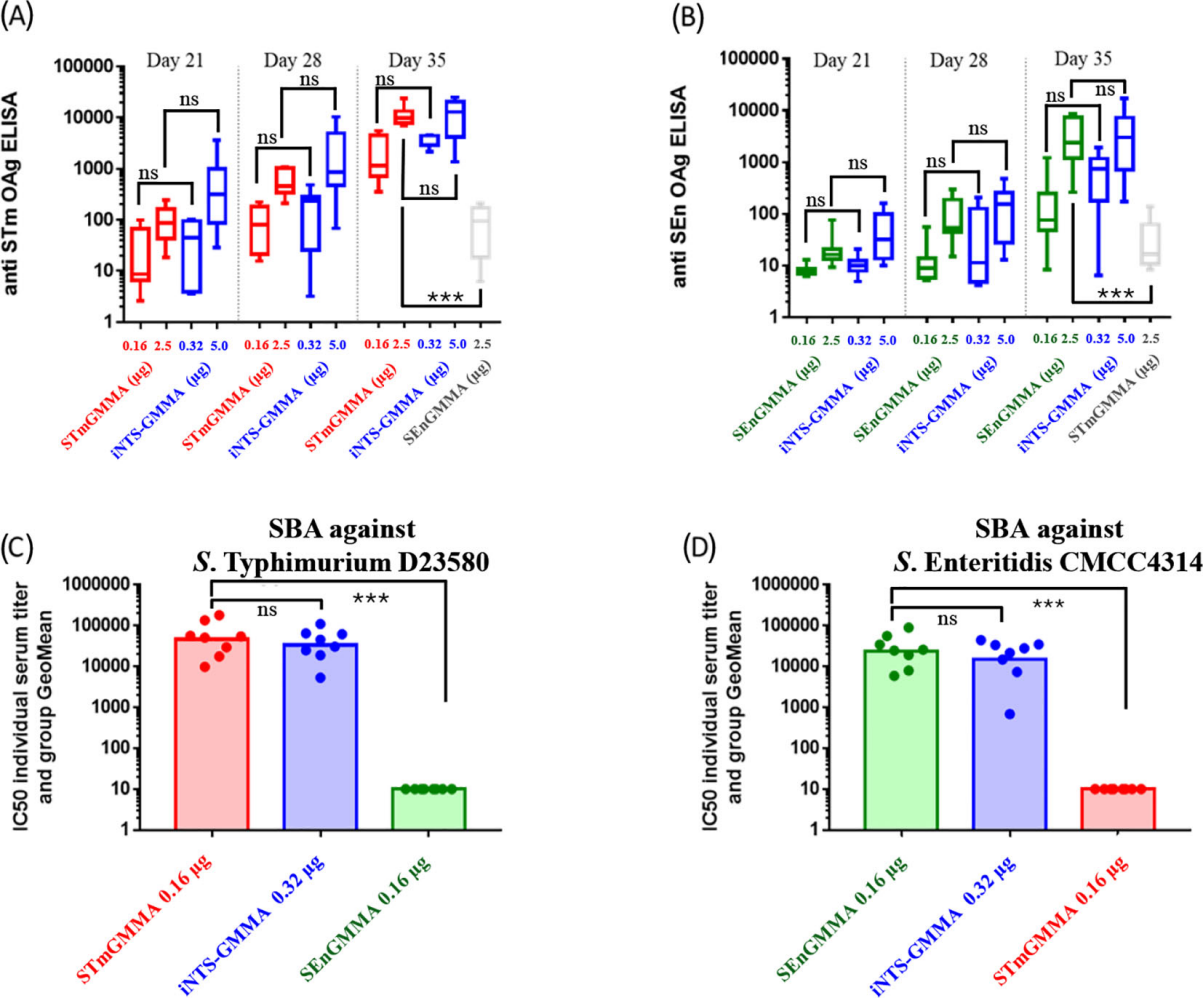
Comparison of the specific IgG levels and SBA activity of antisera from mice immunized with the individual STmGMMA/Alhydreogel, SEnGMMA/Alhydrogel or the mixture of the two vaccine components (iNTS-GMMA vaccine). Antigen-specific antibody distribution in groups immunized with STm or SEn GMMA formulated on Alhydrogel and iNTS-GMMA vaccine assessed by ELISA and results expressed as EU/mL with EU defined as the reciprocal serum dilution resulting in OD = 1 when run in a standard assay. Mice were immunized intraperitoneally on days 0 and 28 and serum collected on days 21, 28 (prior to vaccine injection) and 35 (final bleed). OAg purified from *S.* Typhimurium **(A)** or *S.* Enteritidis **(B)** was used as coating antigen. Concentration of STm and SEn GMMA in the formulations was 0.16 µg and 2.5 µg in OAg either administered as a single components (respectively red and green) or at the same concentration as part of a mixture of formulated components (iNTS-GMMA vaccine, blue) box plots to visualize the distribution of the minimum, first quartile, median (line in the center), third quartile and maximum are shown. A non-parametric Mann-Whitney test was conducted to demonstrate the absence of statistical difference of the response between the single components and the mixture of formulated components (iNTS-GMMA vaccine). P-value annotation *ns* refers to p > 0.05. At bleed day 35, a comparison of titers with heterologous sera with respect to the coating OAg from the single component shows a statistically lower response compared to homologous sera from the single components or from the iNTS-GMMA vaccine (p ≤ 0.001 represented as ***). SBA (IC50) of day 35 sera from mice immunized with 0.16 µg STmGMMA or 0.16 µg SEnGMMA formulated on Alhydrogel or 0.32 µg iNTS-GMMA (0.16 µg STmGMMA/Alhydrogel mixed with 0.16 µg SEnGMMA/Alhydrogel) on day 35. Bactericidal antibody responses against the homologous strain *S*. Typhimurium D23580 **(C)** or *S*. Enteritidis CMCC4314 **(D)** are shown. A non-parametric Mann-Whitney test was conducted to demonstrate the absence of statistical difference between the SBA activity of antisera raised against the single components and the mixture of formulated components (iNTS-GMMA vaccine). P-value annotation *ns* refers to p > 0.05. Absence of significant cross-reactivity is shown by performing SBA with heterologous sera. Statistically lower SBA titers compared to homologous sera from the single component or from the iNTS-GMMA vaccine were identified by Mann-Whitney test (p ≤ 0.001***). Bar charts showing the IC50 of the individual serum titer and the group geometric mean are presented.

In mice, STmGMMA/Alhydrogel or SEnGMMA/Alhydrogel alone or combined into iNTS-GMMA, elicited high levels of specific anti-*S*. Typhimurium OAg or/and anti-*S*. Enteritidis OAg serum IgG in a dose-dependent manner. No difference, as measured by ELISA, was observed when the immunogens were administered as the single vaccine component or as part of the iNTS-GMMA vaccine (**Figures 1A, B**). The antisera had strong *in vitro* bactericidal functionality against homologous *S. enterica* strains (**Figures 1C, D**). No negative immunologic interference was observed in either ELISA or SBA when comparing the single vaccine components versus the iNTS-GMMA vaccine.

Immunogenicity in mice, as a measure of vaccine component immuno-potency, comparing freshy prepared reference standards versus toxicology lots stored at 2-8 °C was carried out for the STmGMMA and SEnGMMA formulations. Results of the potency study at 6 months of the toxicology lots STmGMMA/Alhydrogel and SEnGMMA/Alhydrogel compared with the freshly formulated reference standards are shown as an example of the potency assay (**Supplementary Figure 4**). There was a highly significant dose response (Spearman rank *P* < 0.001 for all the components) with each toxicology lot after one and two immunizations on days 27 and 42, respectively. Linear regression of the log-transformed ELISA units versus the log-transformed doses showed similar slopes and Y-intercepts for the reference standards and toxicology lots for each component. Thus, there was no detectable loss of potency on storage at 2-8°C for this time point (**Supplementary Figure 5**). In conclusion, over the 42 months of stability assessment, no loss of immunogenicity was identified using an *in vivo* potency test that showed no statistically different antibody responses to *S*. Typhimurium and *S*. Enteritidis OAg of the STmGMMA/Alhydrogel and SEnGMMA/Alhydrogel toxicology lots and GMP lots compared to the responses of freshly produced reference standards.

### 3.5 Immunogenicity in rabbits

**Figure 2:**
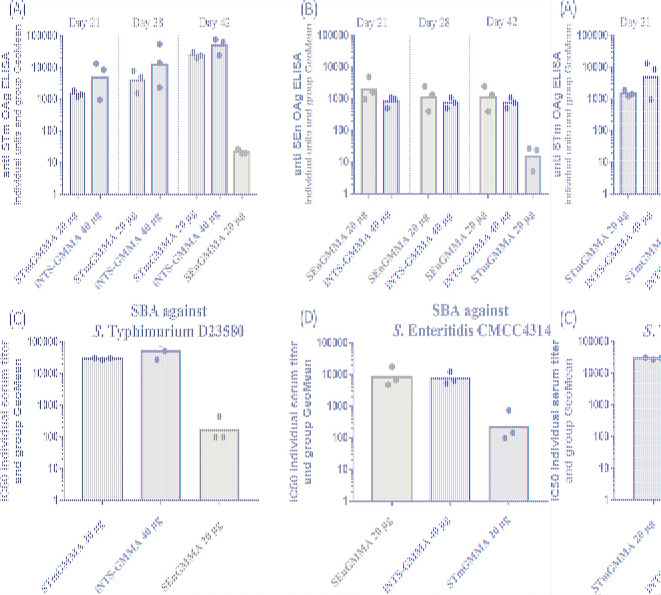
Anti-*S.* Typhimurium and *S.* Enteritidis OAg IgG antibody responses and SBA activity of antisera from rabbits immunized with STmGMMA/Alhydrogel, SEnGMMA/Alhydrogel or the iNTS-GMMA vaccine. Individual anti-*S*. Typhimurium **(A)** and anti-*S.* Enteritidis **(B)** OAg IgG ELISA units measured in sera from rabbits immunized with STmGMMA/Alhydrogel, SEnGMMA/Alhydrogel or their prior to injection mixture (iNTS-GMMA vaccine) at three time points. Graph showing individual antibody titers (and geometric mean bars for graphical proposes). SBA (IC50) of day 42 sera from rabbits immunized with STmGMMA/Alhydrogel, SEnGMMA/Alhydrogel or their prior to injection mixture (iNTS-GMMA vaccine) against the homologous serotype target bacteria *S*. Typhimurium D23580 **(C)** or *S*. Enteritidis CMCC4314 **(D)** (absence of significant cross-reactivity is shown by performing SBA with heterologous sera, which did not elicited SBA activity). The rabbits were vaccinated intramuscularly on day 0 and day 28 with iNTS-GMMA vaccine containing 20 µg of STm OAg GMMA and 20 µg of SEn OAg GMMA (40 µg OAg [total]/dose) or with single component formulations containing an equivalent amount of each OAg GMMA on Alhydrogel. Serum samples were collected on days 21, 28 (prior to vaccine injection) and 42 (final bleed). Bar charts showing the IC50 of the individual serum titer and the group geometric mean are presented.

The iNTS-GMMA vaccine candidate elicited a strong anti-*S.* Typhimurium and *S.* Enteritidis OAg IgG response in rabbits and the response was not inferior compared with the response of the individual STmGMMA/Alhydrogel or SEnGMMA/Alhydrogel vaccine components, respectively. This assessment used the non-GMP reference lots produced at GVGH. Rabbits (N = 3 per group) were immunized intramuscularly on days 0 and 28 with the maximum envisioned human dose of the bivalent iNTS-GMMA vaccine candidate or an equivalent amount of the individual STmGMMA/Alhydrogel and SEnGMMA/Alhydrogel formulations. No immunological interference was observed against homologous OAg by combining the two components into the iNTS-GMMA vaccine (**Figures 2A, B**). These sera showed strong serum bactericidal activity, with similar magnitude of the functional response observed between the sera raised against the two individual vaccine components and the sera raised against the bivalent vaccine mixture (**Figures 2C, D**).

### 3.6 Intramuscular pyrogenicity test in rabbit and its substitution with MAT to assess the relative pyrogenic units of the bivalent iNTS-GMMA vaccine candidate

A pyrogenicity study was performed in rabbits following intramuscular administration of the bivalent iNTS-GMMA vaccine candidate as well as the single component STmGMMA/Alhydrogel and SEnGMMA/Alhydrogel non-GMP lots produced at GVGH. The group mean maximum temperature rise of rabbits that received the bivalent iNTS-GMMA vaccine candidate was 39.7 °C and returned to baseline at the end of the experiment. A similar trend was observed in a group of 3 rabbits that received the individual STmGMMA/Alhydrogel or SEnGMMA/Alhydrogel vaccine components (**Table 2**). In addition, two control groups of 3 rabbits each, received either saline or Alhydrogel diluent in the same study. The group mean maximum temperature rise in rabbits injected with saline was 0.1 °C, meeting the negative control group criterion (≤0.3 °C), and the pyrogenicity test was considered valid. The Alhydrogel diluent group had a mean maximum temperature rise of 0.3 °C. The pyrogenicity profile of the iNTS-GMMA vaccine candidate was considered acceptable, returning to baseline within a 42-hour timeframe and with a maximum temperature increase not exceeding 41 °C. The measured temperature rise of each rabbit at time intervals up to 5 h after vaccination with the iNTS-GMMA vaccine candidate is shown in **Table 2**. Because the fever response was transient, of acceptable magnitude, and accompanied by overall good tolerability in the rabbit pyrogenicity test, a GLP toxicology study was subsequently performed.

**Table 2:** Results of pyrogenicity test of undiluted single GMMA Alhydrogel formulations and bivalent iNTS-GMMA vaccine in rabbits after intramuscular administration.

Administered formulations, (doses based on OAg)	Group average initial temp. [°C]	Group mean maximum temperature [°C]	Group mean temperature [°C] at end of measurement [5 h after vaccination]	Time of mean peak temperature after vaccination [minutes]	Mean peak temperature rise [°C]
STmGMMA/Alhydrogel 20 μg (batch BPR-6075)	38.6	39.7	39.5	180-240	1.2
SEnGMMA/Alhydrogel 20 μg (batch BPR-6076)	38.8	39.7	39.7	240-300	0.9
iNTS-GMMA (STmGMMA/Alhydrogel batch BPR-6075 + SEnGMMA/Alhydrogel batch BPR-6076) 20 μg + 20 μg	38.6	39.7	39.6	210-270	1.1

We additionally characterized the STmGMMA or SEnGMMA and the bivalent iNTS-GMMA vaccine in a MAT assay that was set up with the aim of replacing the pyrogenicity test. Reduced release of IL-6 by human PBMCs was demonstrated by the GMMA produced under GMP conditions from the vaccine *S*. Typhimurium and *S*. Enteritidis production strains in a MAT assay (**Supplementary Figure 2B**) using a previously described methodology (17). These GMP GMMA were used as comparator to calculate the relative pyrogenic unit (RPU) of the bivalent iNTS-GMMA vaccine in a MAT assay performed following Ph. Eur. Chapter 2.6.30 Method 2. This assay was introduced in the panel of characterization of the iNTS GMMA vaccine lots in substitution of the pyrogenicity test for clinical batches as deemed more appropriate to monitor the pre-clinical safety profile of the product. At this stage of the vaccine development, it was decided to consider this test for additional characterization only, without a target development range, and collect more data to set limits based on the experience gained in clinical trials. The RPU of the samples and the geometric coefficient of variation (GCV) of the different STmGMMA/Alhydrogel and SEnGMMA/Alhydrogel GMP lots as bivalent iNTS-GMMA vaccine were calculated against the reference lots and are reported in **Table 1B**.

### 3.7 Repeat dose toxicity study in rabbits

For the repeat dose toxicity study, rabbits were selected as the appropriate animal species, as accepted by regulatory authorities, because historical control data were available, the full human dose (0.5 mL) could be administered by the intended clinical route of administration (IM), and preliminary immunogenicity studies showed the expected immune response, confirming suitability for this study. The bivalent iNTS-GMMA vaccine candidate, prepared by mixing the single components STmGMMA/Alhydrogel and SEnGMMA/Alhydrogel immediately prior to injection, was administered to rabbits at the highest anticipated human dose (i.e. 40 μg total; 20 μg OAg from each component in a final volume of 0.5 mL) as four intramuscular injections given two weeks apart (i.e. days 1, 15, 29 and 43). Necropsies were performed on half the rabbits 3 days (main necropsy, i.e. day 46) or 28 days (recovery necropsy, i.e. day 72) after the last vaccine immunization. The iNTS-GMMA vaccine was systemically well tolerated. No mortality, or treatment-related clinical signs were observed, and no notable changes in body weight or food consumption occurred in treated rabbits. Locally, iNTS-GMMA induced very slight edema and erythema at the dosing sites after two or more injections, findings that are commonly associated with the expected immune and inflammatory responses following immunization with similar vaccines. Temporary elevations in body temperature were noted only at 2 and 6 h post each dose. Increased inguinal and iliac draining lymph node weights and weight ratios were also seen. Microscopically, the dosing sites showed interstitial granulomatous inflammatory cell infiltrates and the inguinal and/or iliac lymph nodes showed signs of hyperplasia at the main necropsy. The microscopic findings at the dosing sites indicated minor, secondary local effects and a reaction consistent with the expected response to the intramuscular iNTS-GMMA vaccine administration; therefore, these findings were considered not adverse. After a 4-week recovery phase, iliac (both sexes) and inguinal (males) lymph nodes, weights and weight ratios were still increased, no macroscopic findings were observed, and microscopic findings of granulomatous inflammatory cell infiltrates at the dosing sites and hyperplasia of the inguinal and/or iliac lymph nodes persisted.

Findings from the repeat dose toxicology study concluded that the bivalent iNTS-GMMA vaccine candidate injected four times, two weeks apart, to both male and female rabbits at the highest anticipated human dose was systemically well tolerated.

**Figure 3:**
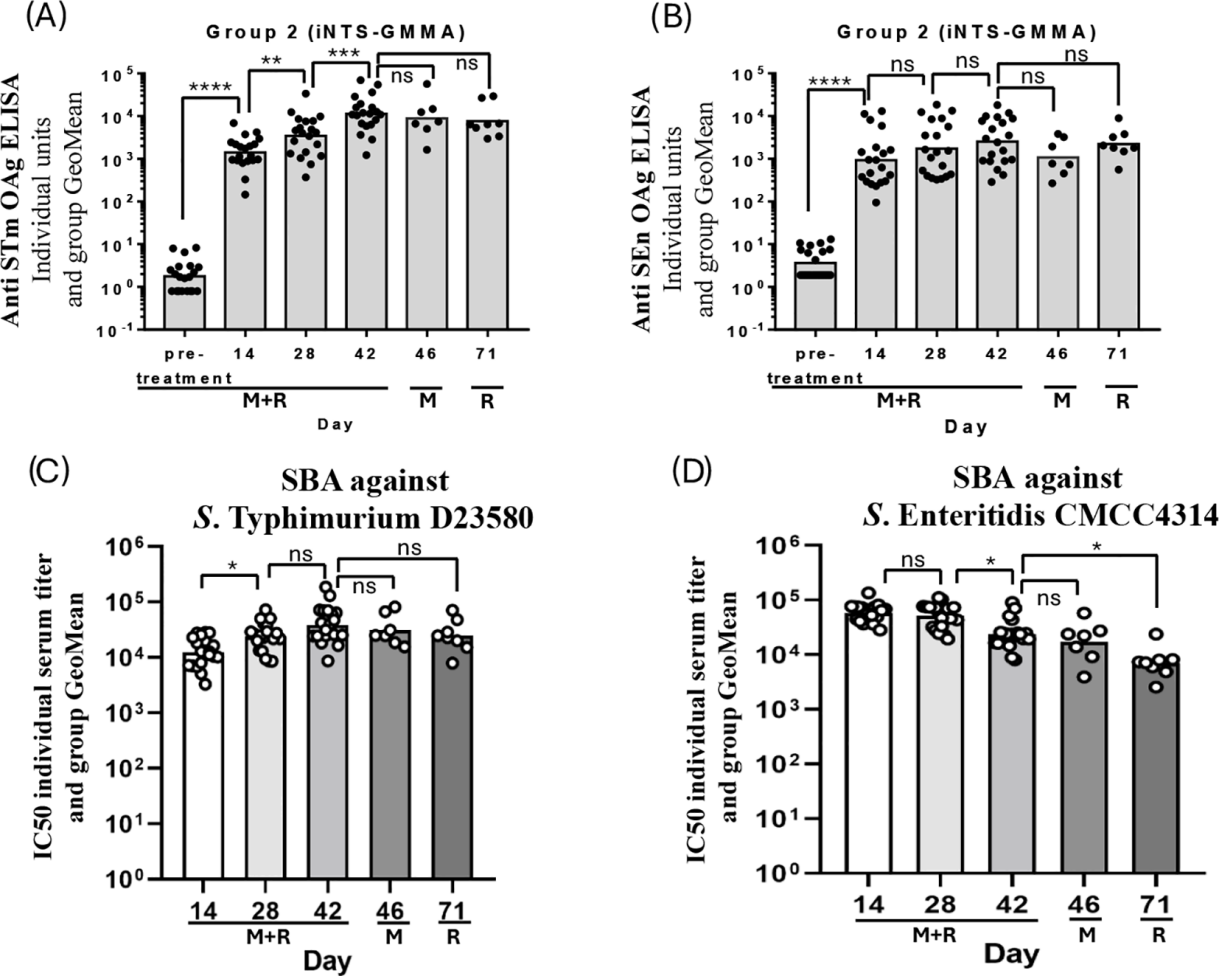
Anti-*S.* Typhimurium and *S.* Enteritidis OAg IgG antibody responses and SBA activity of antisera from rabbits after immunization with the iNTS-GMMA vaccine as part of toxicology study. Graphs show the anti-STm **(A)** or anti-SEn **(B)** OAg IgG responses measured in individual rabbits immunized with iNTS-GMMA (20 µg STmGMMA/Alhydrogel toxicology lot R-0007–02 mixed with 20 µg SEnGMMA/Alhydrogel toxicology lot R-0007-01) (named Group 2 in the toxicology study). Rabbits were immunized on days 1, 15, 29 and 43, and results with sera obtained at pretreatment and on days 14, 28, 42, 46 and 71 are shown. SBA titers (IC50) measured in individual rabbits immunized with the iNTS-GMMA vaccine are reported against the homologous serotype target bacteria *S*. Typhimurium D23580 **(C)** or *S*. Enteritidis CMCC4314 **(D)**. White circles represent serum samples from individual rabbits. Bars represent the group geometric means. Sera from groups of mice immunized with saline or at pretreatment did not show anti-STm or anti-SEn OAg IgG titers nor bactericidal activity. A non-parametric Wilcoxon matched-pairs signed-rank test was conducted to assess the responses of the rabbits prior to and after the administrations of the iNTS-GMMA vaccine. P-value annotation *ns* refers to p > 0.05, P ≤ 0.05 *, p ≤ 0.01 **, p ≤ 0.001 ***, p ≤ 0.0001 ****. M=Main Animals, animals euthanized at the main necropsy (day 46). R= Recovery Animals, animals euthanized at the recovery necropsy (day 71).

Additionally, the non-GLP immunogenicity phase of the toxicology study confirmed previous findings (**Figure 3**) that the iNTS-GMMA vaccine candidate was highly immunogenic in rabbits when administered via the intramuscular route. Increased anti-*S*. Typhimurium and anti-*S*. Enteritidis OAg serum IgG levels were noted on days 14, 28, 42, 46 and 71 compared with both pre-treatment and concurrent control animal values, indicating a specific antibody response.

## 4 Discussion

The OAg is the main target for vaccines against iNTS disease, and some OAg-based vaccines have reached early clinical development. For *S*. Typhimurium and *S*. Enteritidis, the OAg have been conjugated to carrier proteins such as CRM_197_, flagellin (FliC), or diphtheria toxoid (DT), yielding immunogenic and protective responses in animal models (45). However, multivalent conjugate-based vaccines are technically complex, costly to manufacture, and could limit affordability in LMIC. GMMA represent an alternative vaccine platform technology that may overcome some of these limitations. Importantly, they can be produced at production-scale using relatively simple fermentation and tangential flow filtration processes, which support low cost and technology transfer (46, 47). A previous comparative study directly evaluated bivalent *S.* Typhimurium and *S.* Enteritidis GMMA versus equivalent OAg–CRM_197_ glycoconjugates in mice, using OAg with closely matched structural characteristics in both platforms (23). When formulated with Alhydrogel, GMMA and glycoconjugates induced similar OAg-specific IgG titers; however, GMMA elicited a broader antibody isotype profile, including higher levels of IgG2b, IgG3 and IgM, and consistently higher serum bactericidal activity against both *S.* Typhimurium and *S.* Enteritidis. In a mouse infection model, immunization with either bivalent GMMA or conjugate vaccines led to a significant reduction in bacterial colonization of the spleen and liver after intraperitoneal challenge with African *S*. Typhimurium D23580 or *S*. Enteritidis D24954 strains compared to control mice, but *S.* Typhimurium GMMA conferred a greater reduction in liver bacterial counts than the corresponding conjugate formulation (23). Another study evaluated the impact of STmGMMA/Alhydrogel immunization on a murine intravenous infection model with the *S*. Typhimurium D23580 strain. Immunized mice showed a significantly lower bacterial load in blood, spleen, and liver samples 24 hours after infection compared to saline-treated animals. This reduced bacterial load was also observed in the spleen and liver when mice were challenged 14 weeks after booster immunization. A positive correlation was observed between higher O:4,5 specific IgG in serum and the bacterial CFU in the spleens of immunized and challenged mice, underscoring the protective immune response (24). Although antibody responses are a primary focus in assessing iNTS vaccine immunogenicity, no correlate of protection for iNTS has yet been established. Protection against iNTS disease likely involves a combination of both humoral and cellular immune responses (28, 45). Although GMMA mechanism of action has not been fully characterized, they are likely to activate different pathways of the immune response, as an example Baker and co-authors demonstrated that OMV could induce robust cellular immune responses that exceeded those induced by a live-attenuated strain (48). Traditional vaccines for *Salmonella* often lean on antibodies and humoral response generation based on observations of natural protection correlating with NTS-specific antibodies, and their direct roles in bacterial killing and opsonization (9, 49, 50). However, due to the intracellular nature of *Salmonella*, T-cell immunity will be important for clearance and to effectively produce antibodies, particularly in vulnerable populations where T-cell function can be compromised (11, 12, 28). Taken together, these data indicated that GMMA can match or exceed the immunogenicity and protective efficacy of established glycoconjugate vaccines, while offering a simpler and potentially more affordable manufacturing platform technology.

The bivalent iNTS-GMMA vaccine candidate contains GMMA from the most commonly found *S. enterica* serovars Typhimurium and Enteritidis in sSA, accounting for more than 90% of the iNTS disease cases. We had previously demonstrated antigenic similarity between the OAg from the African *S*. Typhimurium D23580 isolated from human blood and the OAg from *S*. Typhimurium strain 2192 isolated from an animal (30). In this study, *S.* Typhimurium 2192 with *tolR, msbB, pagP* mutations is used for the iNTS-GMMA vaccine candidate due to its optimized and consistent production profile. While earlier studies explored different *S.* Typhimurium parent strains or mutation profiles (17, 23, 51), the GMMA generated by exploratory batches from those strains possessed quality attributes that were considered suboptimal for their selection as vaccine production strains when compared to GMMA generated from the *S.* Typhimurium 2192 triple mutant. *S.* Enteritidis strain 618, however, selected as the parent for the generation of GMMA in the iNTS-GMMA vaccine candidate, has been consistently used across these studies as it did not show similar production challenges.

STmGMMA and SEnGMMA drug substances were produced under non-GMP and GMP settings in high yield with simple and reproducible manufacturing processes. Both GMMA were derived from well characterized MCB prepared from *S*. Typhimurium and *S*. Enteritidis strains with genetically modified lipid A aiming to reduce the risk of reactogenicity in humans. As part of the manufacturing process, a simple two step filtration/purification was adapted from that previously used to produce a *S. sonnei* GMMA vaccine candidate and used to enable a low-cost manufacture. This extends the knowledge, transferability and utility of the generic and low-cost GMMA technology to other potential vaccines urgently needed in LMIC.

Initially, two separate Alhydrogel-formulated GMMA vaccine components have been produced to allow greater flexibility in early development. Depending on the immunogenicity results observed in clinical trials, adjustment of the STmGMMA/Alhydrogel and SEnGMMA/Alhydrogel component ratio may be necessary to define the optimal iNTS-GMMA vaccine composition. The two separate GMMA formulations were designed for compatibility, and a robust mixing protocol allows these studies to be performed in a flexible, quick and cost-conscious way. This approach is supported by strong stability packages showing minimal changes over time in the characterization of quality attributes of the two Alhydrogel GMMA formulations.

The STmGMMA/Alhydrogel and SEnGMMA/Alhydrogel vaccine components and the iNTS-GMMA vaccine candidate were found to be highly immunogenic in both mice and rabbits. Low doses (in the ng range of OAg) of the single GMMA formulations or the mixture induced high levels of anti-*S*. Typhimurium and *S*. Enteritidis OAg antibodies. Vaccination of mice with only 0.16 µg (OAg) of GMMA from *S*. Typhimurium and *S*. Enteritidis induced substantial serum antibodies after a single injection that boosted following a second injection. We demonstrated that there was no statistically significant difference between the IgG titers in sera or the antibody functionality (as determined by SBA) of sera raised from the individual vaccine components compared with the iNTS-GMMA vaccine. This demonstrated that, at the tested concentrations, the mixture of STmGMMA/Alhydrogel and SEnGMMA/Alhydrogel did not elicit immuno-interference and one component was not immuno-dominant over the other.

The observed immunogenicity in mice following a single injection facilitated the development of a potency test, as part of the stability assessment of the drug products over time. The selected dose range allows direct comparison of relative immunogenicity of STmGMMA/Alhydrogel and SEnGMMA/Alhydrogel vaccine components to freshly formulated reference standards. This potency assay, together with biochemical stability assessment of the Alhydrogel-formulated STmGMMA and SEnGMMA drug products, allowed the shelf-life assignment of up to 42 months at 2–8 °C based on current data.

Pre-clinical safety assessment of the iNTS-GMMA vaccine candidate and its components supported Phase I testing. The GMP-produced STmGMMA and SEnGMMA showed reduced IL-6 induction *in vitro* from human PBMC and the level of IL-6 was comparable to another GMMA-based candidate vaccine tested in humans (16, 17, 32). *In vivo* fever response in rabbits following intramuscular administration of full human dose of the iNTS-GMMA vaccine candidate was considered acceptable using a modified rabbit pyrogenicity model. No observation of concern was reported in a GLP repeat dose rabbit toxicology study of the iNTS-GMMA vaccine candidate.

The manufacturability process proved robust and the STmGMMA and SEnGMMA drug substances, together with the STmGMMA/Alhydrogel and SEnGMMA/Alhydrogel drug products, demonstrated stability over time. These data, along with the immunogenicity responses observed in mice and rabbits by these iNTS-GMMA vaccine components, and the acceptable safety profile from the *in vitro* and *in vivo* analyses, supported progression to clinical studies. Thus, the bivalent iNTS-GMMA vaccine candidate has the potential to be an affordable and effective vaccine to address a major invasive bacterial disease of infants in sSA.

## The Vacc-iNTS consortium collaborators

Francis Agyapong (Kwame Nkrumah University of Science and Technology Kumasi). Gianluca Breghi (Fondazione Achille Sclavo). Annalisa Ciabattini (University of Siena). John A. Crump (University of Otago). Melita A Gordon (University of Liverpool). Samuel Kariuki (Kenya Medical Research Institute). Brama Hanumunthadu (University of Oxford). Liselotte Hardy (Institute of Tropical Medicine Antwerp). Stefano Malvolti (MM Global Health Consulting). Carsten Mantel (MM Global Health Consulting). Christian S. Marchello (University of Otago). Florian Marks (University of Cambridge and International Vaccine Institute). Donata Medaglini (Università di Siena). Tonney S. Nyirenda (University of Malawi). Mercy Ngetich (Kenya Medical Research Institute). Elena Pettini (University of Siena). Ellis Owusu-Dabo (Kwame Nkrumah University of Science and Technology Kumasi). Maheshi N. Ramasamy (University of Oxford). J. Anthony G. Scott (KEMRI-Wellcome Trust Research Programme). Bassiahi Abdramane Soura (University of Ouagadougou). Tiziana Spadafina (Sclavo Vaccines Association). Bieke Tack (Institute of Tropical Medicine Antwerp).

## Data Availability

The original contributions presented in the study are included in the article/**Supplementary Material**. Further inquiries can be directed to the corresponding author.
