# Supplementary material for: Development of a GMMA-based candidate vaccine against invasive nontyphoidal Salmonella disease
Source: Front Immunol. 2026 Jun 2;17:1821495. doi: 10.3389/fimmu.2026.1821495 (PMC13270084; doi:10.3389/fimmu.2026.1821495)
Supplement: Supplementary file 1 [file Table1.docx]

Supplementary Material

# Supplementary Figures and Tables

## Supplementary Figures

**Supplementary Figure 1.**

**A**

**
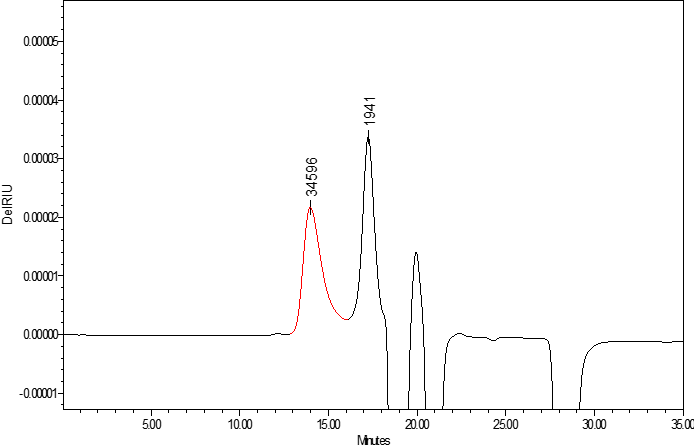
**

**B**

**Supplementary Figure 1.** HPLC-SEC of OAg extracted from STmGMMA (**A**) and SEnGMMA (**B**) generated from vaccine production strains. OAg molecular weights of medium population (red) and low population are shown.

**Supplementary Figure 2.**

**(A)**

**
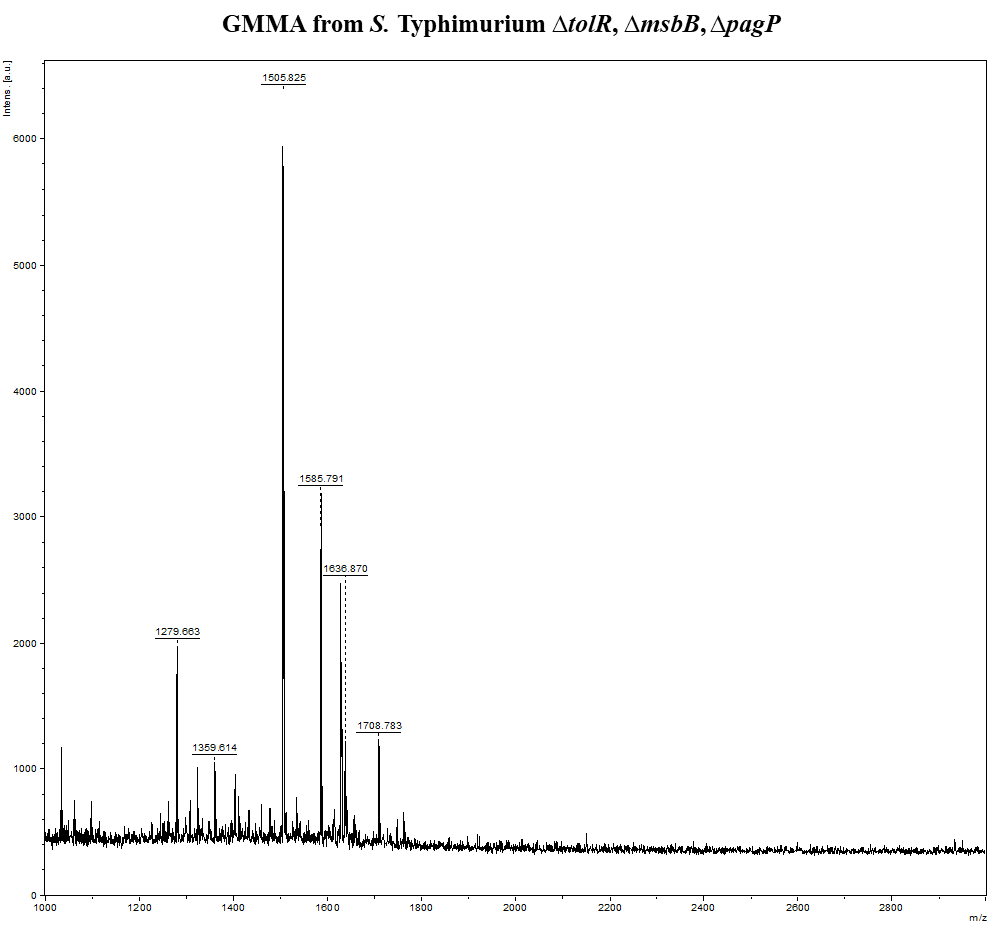
**

Pentaacylated Lipid A (– phosphate group)

Pentaacylated Lipid A + phosphoethanolamine

Pentaacylated Lipid A + Arabinosammine - Phosphate

Pentaacylated Lipid A

**
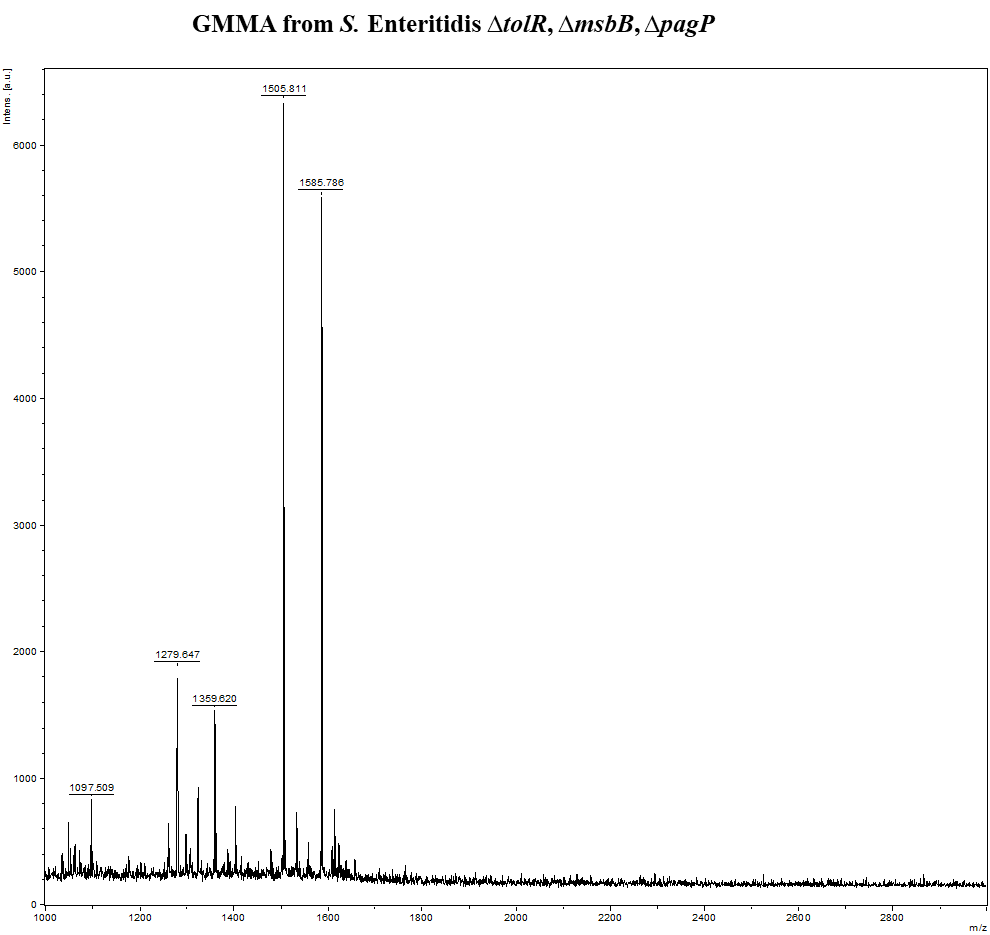
**

Pentaacylated Lipid A

Pentaacylated Lipid A (– phosphate group)

**(B)**

**
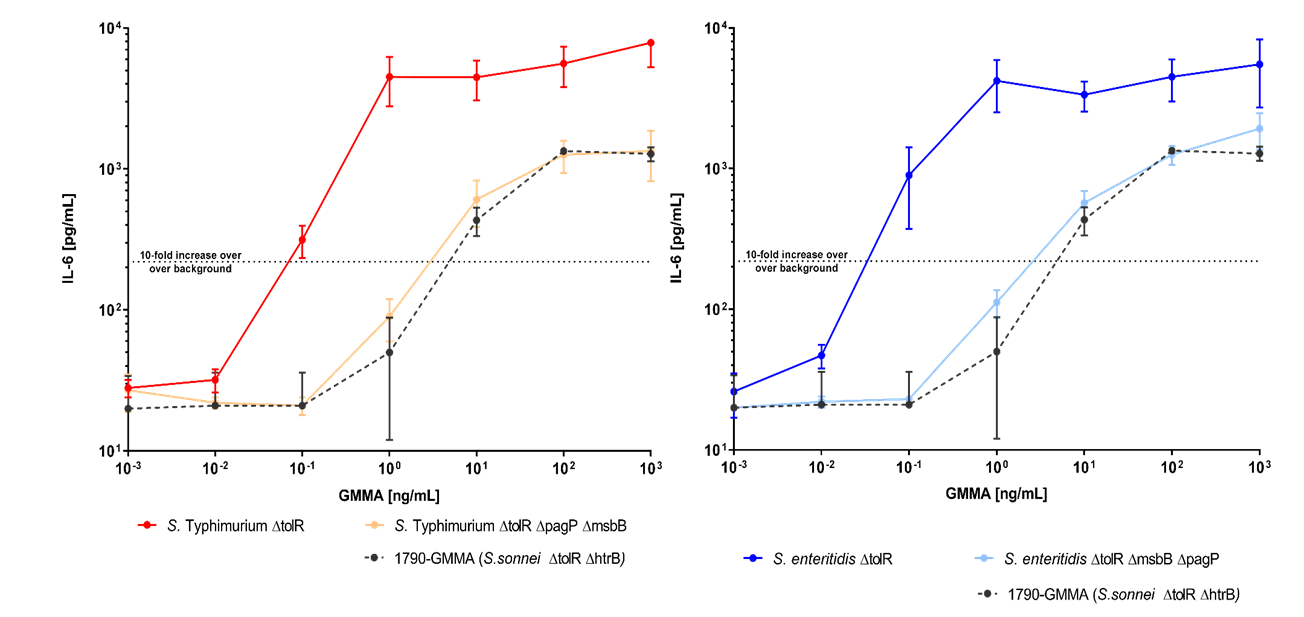
**

**Supplementary Figure 2.**

**(A)** Mass spectroscopy using MALDI-TOF for analysis of lipid A extracted from GMMA from *S.* Typhimurium ∆*tolR*, ∆*msbB*, ∆*pagP* and *S*. Enteritidis ∆*tolR*, ∆*msbB*, ∆*pagP* produced from the GMMA production strains carrying a modified lipid A compared to the wild-type lipid A. The structure showed a predominant peak at 1585 m/z consistent with a lipid A lacking a myristoyl chain [39]) and minor peaks due to fragmentation (i.e. loss of one or more fatty acid chains), aggregate formation with sodium (+23 m/z) or de-phosphorylation (-80 m/z). The MALDI-TOF profile of lipid A purified from the wild-type *S.* Typhimurium or *S.* Enteritidis had the expected lipid A structure with hepta- or hexa-acylated species as shown by peak at 1796 m/z as previously reported [19].

**(B)** MAT Assay
In the human MAT, different concentrations based on protein content of STm GMMA from the vaccine production strain (*S.* Typhimurium ∆*tolR* ∆*msbB* ∆*pagP*) or SEnGMMA from the vaccine production strain (*S.* Enteritidis ∆*tolR* ∆*msbB* ∆*pagP*) stimulate similar release of IL-6 to the corresponding concentrations (in proteins) of 1790-GMMA (*S. sonnei* ∆*tolR* ∆*msbB*). All genetically modified GMMA require approximately 100–fold higher concentration to give the same IL-6 production as STmGMMA or SEnGMMA with a wild-type lipid A (∆*tolR* strains). IL-6 released by human PBMC was measured after 4-hour incubation with GMMA. Each curve shows the IL6 release data points obtained stimulating PBMC with serial dilution of GMMA assayed in technical duplicates. Average of IL-6 release by PBMC for each GMMA concentration assayed is shown in symbols. The horizontal dashed line is the approximate 10-fold over background IL-6 level. The assay was repeated using PBMC from 3 different donors; each PBMC donor was assayed twice (biological replicates) in duplicate (technical replicates). One representative experiment is shown.

**Supplementary Figure 3.**


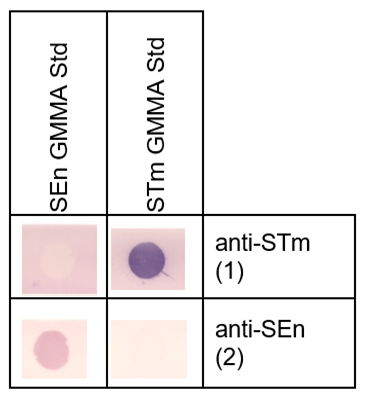


**Supplementary Figure 3.** Dot blot of SEnGMMA and STmGMMA using commercially available typing antibodies; the strip 1 was tested using STm-specific monoclonal antibody, the strip 2 was tested using SEn-specific monoclonal antibody.

**Supplementary Figure 4**
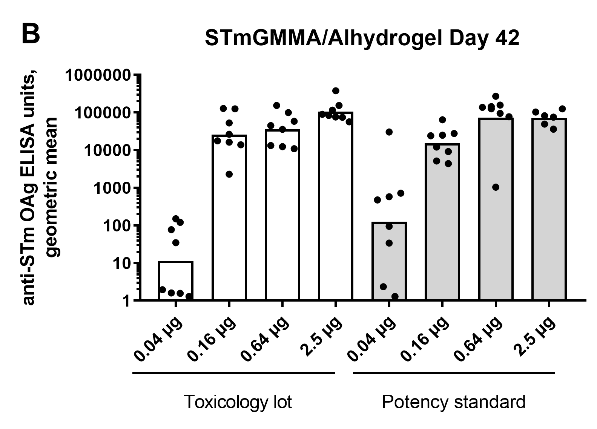
**
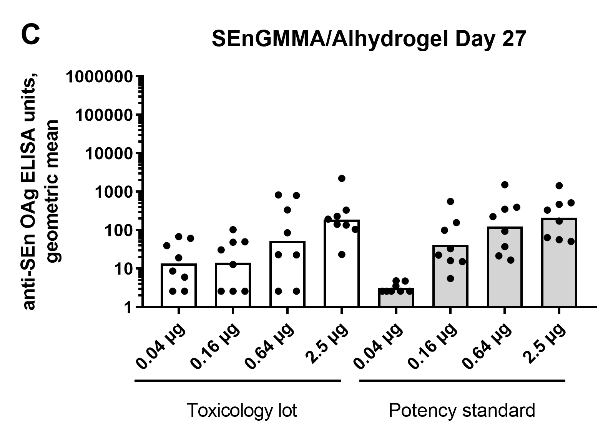
**
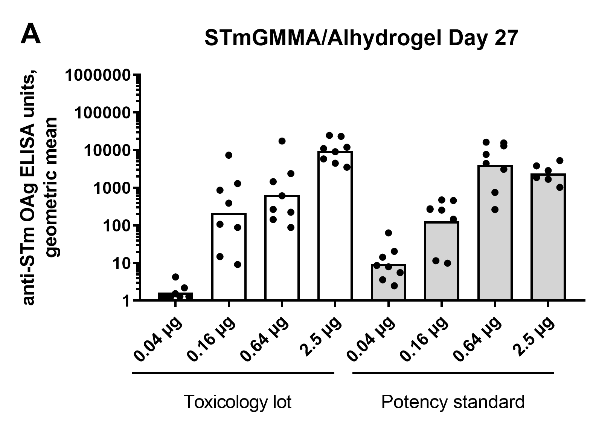
**.**

**
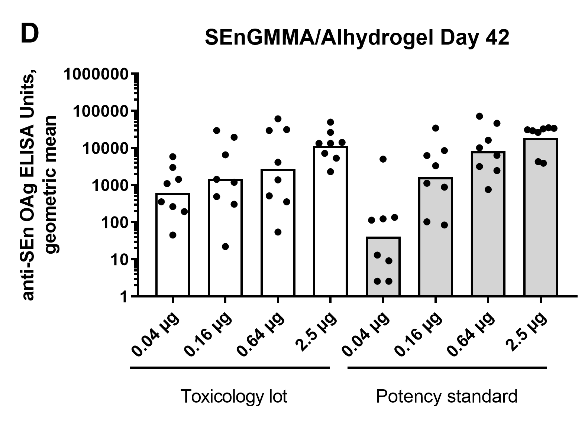
**

**Supplementary Figure 4.** Anti-STm OAg and anti-SEn OAg IgG antibody responses in mice after immunization with toxicology lots (STmGMMA/Alhydrogel, SEnGMMA/Alhydrogel) stored at 2-8°C for six months and the respective freshly formulated potency standards.

The graphs show the anti-STm OAg IgG ELISA units measured in individual mice immunized with 0.04 µg, 0.16 µg, 0.64 µg and 2.5 µg OAg of STmGMMA/Alhydrogel toxicology lot (white bars) or the STmGMMA/Alhydrogel potency standard (grey bars) on days 27 (**A**) or 42 (**B**). Graphs showing the anti-SEn OAg IgG ELISA units measured in individual mice immunized with 0.04 µg, 0.16 µg, 0.64 µg and 2.5 µg OAg of SEnGMMA/Alhydrogel toxicology lot (white bars) or the SEnGMMA/Alhydrogel potency standard (grey bars) on days 27 (**C**) or 42 (**D**). Symbols represent results from individual mice. Bars represent group geometric mean ELISA units. Mice were immunized intraperitoneally on days 0 and 28 with STmGMMA/Alhydrogel or SEnGMMA/Alhydrogel. A single IP immunization of groups of 8 mice with sera analyzed at day 27 was selected for use as vaccine potency assessment at release and as part of stability.

**Supplementary Figure 5**


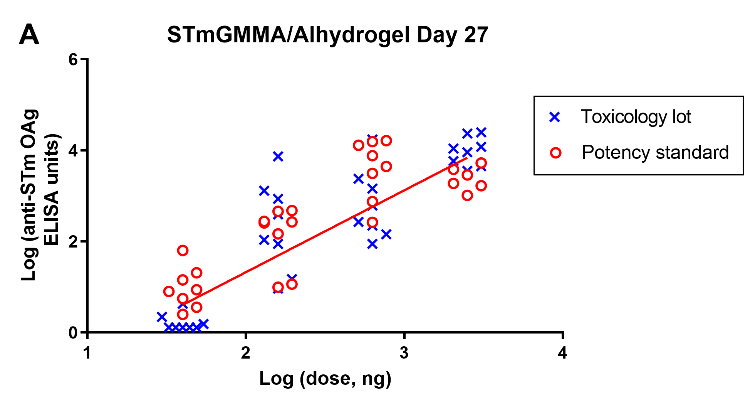

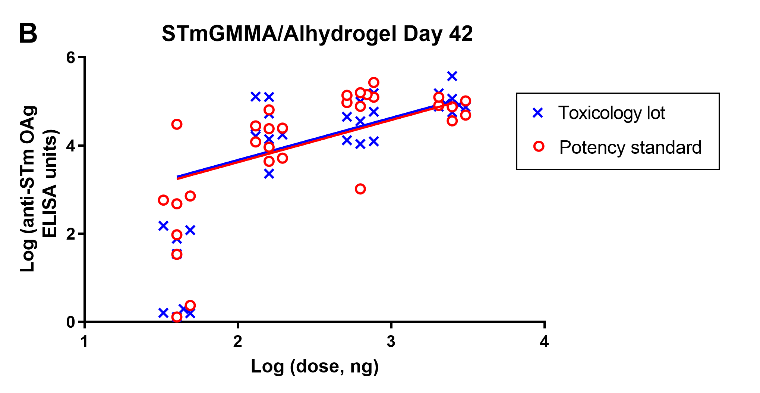


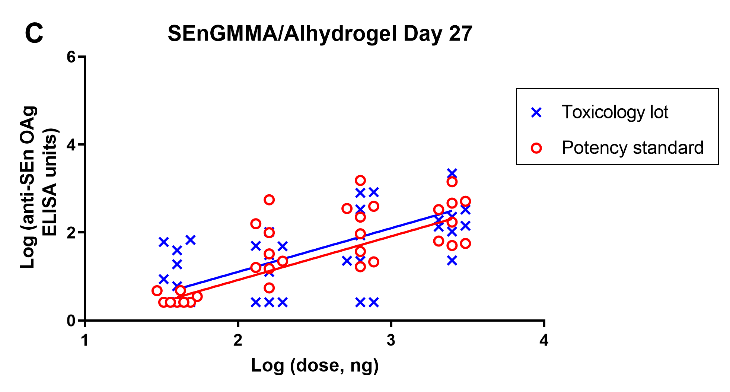

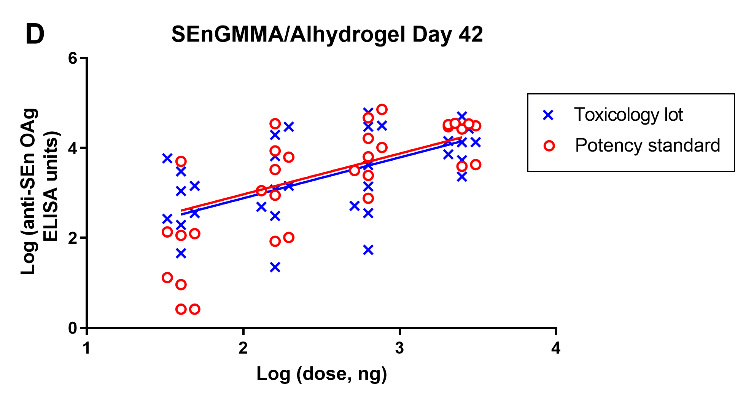


**Supplementary Figure 5**

Analysis of the anti-STm OAg and anti-SEn OAg IgG antibody responses in mice after immunization with toxicology lots (STmGMMA/Alhydrogel, SEnGMMA/Alhydrogel) stored at 2-8°C for six months and the respective freshly formulated potency standards.

The Scatter plots show the log-transformed individual results of the anti-STm OAg and anti-SEn OAg IgG responses in sera obtained after 27 days and 42 days from mice immunized with the toxicology lots (STmGMMA/Alhydrogel and SEnGMMA/Alhydrogel) stored for six months at 2-8°C and the respective freshly formulated potency standards.

## Supplementary Tables

**Supplementary Table 1.**

**Differences between DM used for fermentation of *S*. Enteritidis or *S*. Typhimurium and *Shigella sonnei* DM**

| **1000X Trace Elements Solution** | **SEn DM** | **STm DM** | ***S. sonnei* DM** |
| --- | --- | --- | --- |
| Sulfuric Acid H2SO4 | 20 mL/kg | 20 mL/kg | 20 mL/kg |
| Manganese chloride tetrahydrate MnCl2 · 4H2O | 15 g/kg | 15 g/kg | 15 g/kg |
| Copper Chloride dihydrate CuCl2 · 2H2O | 1.5 g/kg | 1.5 g/kg | 1.5 g/kg |
| Boric Acid solution 4% H3BO3 | 75 g/kg | 75 g/kg | 75 g/kg |
| Sodium Molybdate Dihydrate Na2MoO4*2H2O | 2.5 g/kg | 2.5 g/kg | 2.5 g/kg |
| Zinc acetate dihydrate Zn(CH3COO)2 · 2H2O | 13 g/kg | 13 g/kg | 13 g/kg |
| Hexammine cobalt(III) chloride [Co(NH3)6]Cl3 | 2.5 g/kg | 2.5 g/kg | 2.5 g/kg |
| **1000X Ferric citrate** | **SEn** | **STm** | ***S. sonnei*** |
| Ferric Citrate  C6H5FeO7 | 24.5 g/kg | 24.5 g/kg | 12.5 g/kg |
| **500X Magnesium Sulfate Solution** | **SEn** | **STm** | **S. Sonnei** |
| Magnesium Sulfate heptahydrate MgSO4*7H2O | 246.5 g/kg | 246.5 g/kg | 246.5 g/kg |
| 1000X Vitamins Solution |  |  |  |
| Thiamine Hydrochloride C12H17ClN4OS*HCl | 50 g/kg | 50 g/kg | 50 g/kg |
| NIcotinic Acid C6H5NO2 | 10 g/kg | 10 g/kg |  |
| Piridoxine-HCl  C8H11NO3 · HCl | 10 g/kg | 10 g/kg |  |
| Calcium-pantothenate  C18H32CaN2O10 | 10 g/kg | 10 g/kg |  |
| Cyanocobalamin  C63H88CoN14O14P | 1 g/kg | 1 g/kg |  |
| **Complete Inoculum medium** | **SEn** | **STm** | ***S. sonnei*** |
| Potassium Phosphate Monobasic KH2PO4 | 13.3 g/kg | 13.3 g/kg | 13.3 g/kg |
| Di-Ammonium HydrogenPhosphate (NH4)2HPO4 | 4 g/kg | 4 g/kg | 4 g/kg |
| Citric Acid Monohydrate C6H8O7*H2O | 1.7 g/kg | 1.7 g/kg | 1.7 g/kg |
| Dextrose monohydrate C6H12O6 · H2O | 10 g/kg | 10 g/kg | 5 g/kg |
| L-Hystidine  C6H9N3O2 | 0.22 g/kg |  |  |
| L-Serine HOCH2CH(NH2)CO2H | 0.28 g/kg |  |  |
| L-Phenylalanine  C6H5CH2CH(NH2)CO2H | 0.62 g/kg |  |  |
| L-Methionine  CH3SCH2CH2CH(NH2)CO2H | 0.16 g/kg | 0.16 g/kg | 0.16 g/kg |
| L-Asparagine |  |  | 0.06 g/kg |
| L-Threonine  CH3CH(OH)CH(NH2)CO2H | 0.22 g/kg | 0.22 g/kg | 0.22 g/kg |
|  |  |  |  |
| L-Arginine  H2NC(=NH)NH(CH2)3CH(NH2)CO2H | 0.56 g/kg | 0.56 g/kg |  |
| L-Aspartic Acid  HO2CCH2CH(NH2)CO2H | 1 g/kg | 1 g/kg | 2.5 g/kg |
| L-Proline  C5H9NO2 | 0.4 g/kg | 0.4 g/kg |  |
| L-Lysine |  |  | 0.6 g/kg |
| L_Glutamic acid monosodium salt  NaOOCCH2CH2CH(NH2)COOH · H2O | 1.78 g/kg | 1.78 g/kg | 1.78 g/kg |

**Supplementary Table 2.**

1. **Long term stability data for SEnGMMA/Alhydrogel toxicology lot R-0007-01 stored at 5°C**

| **Test Parameter** | **Spec** | **Time 0** | **6 months** | **9 months** | **12 months** | **18 months** | **24 months** | **36 months** | **P-value*** |
| --- | --- | --- | --- | --- | --- | --- | --- | --- | --- |
| Quantification of OAg by Competitive ELISA (FAcE)^§^ | Report µg OAg/mL | 81.9 | 94.3 | 44.7 | 65.9 | NP | 78.8 | 82.4 | 0.1333** |
| Protein not adsorbed to Alhydrogel - SDS-PAGE, silver staining, densitometry. | < 10% | <10 | <10 | <10 | <10 | <10 | <10 | <10 | NA |
| pH | 6.0 - 7.0 | 6.4 | 6.5 | 6.5 | 6.5 | 6.5 | 6.5 | 6.6 | ***0.0137*** |
| Osmolality | 240 - 360 mOsm/kg | 319 | 314 | 318 | 316 | 316 | 312 | 321 | 0.7723 |
| Additional test introduced during stability study: Protein quantification by µBCA on Drug Product supernatant^§^ | ND  Report μg/mL | NP | NP | NP | NP | NP | 1.5 | 1.8 | NA |

NA: Not applicable.

ND: Not defined.

NP: Not performed.

^§^Performed by GVGH.

*Linear regression analysis was performed and *P*-values with slope significantly different from zero are highlighted in bold.

**FAcE testing of SEnGMMA/Alhydrogel was performed with polyclonal serum at release and at six months. At nine and twelve months, FAcE testing was performed with anti-SEn OAg monoclonal antibody. FAcE was not performed at 18 months, but was performed from 24 months onwards after characterization of the assay. Thus, changes in quantification with time were calculated based on four time points (9, 12, 24 and 36 months) to assess changes in OAg quantification.

1. **Long term stability data for STmGMMA/Alhydrogel toxicology lot R-0007-02 at 5°C**

| **Test Parameter** | **Spec** | **Time 0** | **6 months** | **9 months** | **12 months** | **18 months** | **24 months** | **36 months** | **P-value*** |
| --- | --- | --- | --- | --- | --- | --- | --- | --- | --- |
| Quantification of OAg by Competitive ELISA (FAcE)^§^ | Report µg OAg/mL | 102.0 | 126.4 | 130.0 | 58.8 | 82.9 | 59.0 | 87.9 | 0.2819 |
| Protein not adsorbed to Alhydrogel - SDS-PAGE, silver staining, densitometry | < 10% | < 10 | < 10 | < 10 | < 10 | < 10 | < 10 | < 10 | NA |
| pH | 6.0 – 7.0 | 6.3 | 6.5 | 6.5 | 6.5 | 6.5 | 6.5 | 6.6 | ***0.0389*** |
| Osmolality | 240 - 360 mOsm/kg | 322 | 316 | 320 | 319 | 317 | 319 | 320 | 0.8952 |
| Additional test introduced during stability study: Protein quantification by µBCA on Drug Product supernatant^§^ | Conform | NP | NP | NP | NP | NP | 3.09 | 3.5 | NA |

NA: Not applicable.

NP: Not performed.

^§^Performed by GVGH.

*Linear regression analysis was performed. *P*-values with slope significantly different from zero are highlighted in bold.

1. **Long term stability data for SEnGMMA/Alhydrogel clinical lot SE-20-002 at 5°C**

| **Test Parameter** | **Acceptance criteria** | **Release** | **3 months** | **6 months** | **9 months** | **12 months** | **18 months** | **24 months** | **36 months^&^** | **42 months** | ***^#^P*-value** |
| --- | --- | --- | --- | --- | --- | --- | --- | --- | --- | --- | --- |
| Quantification of total protein (µBCA) | Report µg OAg/mL | 31 | -* | -* | 27 | 26 | 29 | 26 | - | 32 | 0.591 |
| Quantification of OAg by calculation (based on Drug Product total protein and OAg:protein ratio of Drug Substance) | 43-92 µg/mL | 74 | -* | -* | 65 | 62 | 70 | 63 | . | 77 | 0.537 |
| pH | 6.2 – 7.0 | 6.4 | 6.4 | 6.5 | 6.6 | 6.7 | 6.5 | 6.5 | 6.6 | 6.6 | 0.177 |
| Osmolality | 240 - 360 mOsm/kg | 331 | 336 | 330 | 329 | 322 | 325 | 324 | 327 | 332 | 0.576 |
| Quantification of OAg by competitive-ELISA (FAcE)^§^ | Report µg OAg/mL | 57.8 | 58.9 | 82.4 | 62.2 | 70.2 | 83.1 | 70.4 | 78.8 | - | 0.146 |
| Total protein not adsorbed on Alhydrogel (µBCA on DP supernatant^§^ | Report (μg/mL) | 3.58 | -* | -* | 2.83 | 4.05 | 4.52 | 2.94 | 4.30 | - | 0.538 |
| GMMA not adsorbed on Alhydrogel (lipid A) by HPLC-RP/MS^§^ | Report (nmol/mL) | -** | -** | 0.83 | 0.85 | 1.23 | 0.90 | 1.00 | 1.90 | **-** | **0.049** |

^§^Performed by GVGH.

^#^Linear regression analysis was performed and p-values indicate whether slope is significantly different from zero (α = 0.05).

*Quantification of total protein (µBCA) for release was performed 6 months after production. Therefore, stability testing after 3 and 6 months was not performed.

**Test was implemented as part of the surveillance test and stability panel about 6 months after manufacture and was therefore applied for the first time at the 9 months stability time point.

^&^Surveillance analyses at the end of stability were performed at GVGH only at 36 months. Stability panel analyses were shifted to 42 months and were conducted at Northway Biotech (Vilnius, Lithuania).

1. **Long term stability data for STmGMMA/Alhydrogel clinical lot ST-20-001 at 5°C**

| **Test Parameter** | **Acceptance criteria** | **Release** | **3 months** | **6 months** | **9 months** | **12 months** | **18 months** | **24 months** | **36 months^&^** | **42 months** | ***^#^P*-value** |
| --- | --- | --- | --- | --- | --- | --- | --- | --- | --- | --- | --- |
| Quantification of total protein (µBCA) | Report µg OAg/mL | 84* | -* | -* | 81 | 81 | 79 | 69 | - | 71 | **0.029** |
| Quantification of OAg by calculation (based on Drug Product total protein and OAg:protein ratio of Drug Substance) | 41 - 114 µg/mL | 67* | -* | -* | 66 | 65 | 63 | 55 | - | 57 | **0.035** |
| pH | 6.2 – 7.0 | 6.4 | 6.4 | 6.4 | 6.5 | 6.7 | 6.5 | 6.6 | 6.6 | 6.5 | 0.201 |
| Osmolality | 240 - 360 mOsm/kg | 323 | 329 | 319 | 320 | 323 | 318 | 317 | 317 | 326 | 0.577 |
| Quantification of OAg by competitive-ELISA (FAcE)^§^ | Report µg OAg/mL | 107.2 | 94.3 | 93.6 | 86.3 | 80.5 | 83.4 | 89.8 | 92.1 | - | 0.362 |
| Total protein not adsorbed on Alhydrogel (µBCA on DP supernatant)^§^ | Report (μg/mL) | 5.55* | -* | -* | 4.24 | 5.28 | 4.68 | 3.91 | 5.90 | - | 0.869 |
| GMMA not adsorbed on Alhydrogel (lipid A) by HPLC-RP/MS^§^ | Report (nmol/mL) | -** | -** | 0.41 | 0.46 | 0.54 | 0.50 | 0.50 | 0.98 | **-** | **0.025** |
| Particle size distribution  Static Light Scattering^§^ | D[4,3] report in µm  D(90) report in µm | 8.5  23.9 | 15.1  32.9 | 9.6  27.6 | 9.0  25.4 | 9.5  27.5 | 9.1  24.0 | 11.0  31.4 | 18.0  45.0 | - | 0.152  **0.046** |

^§^Performed by GVGH.

^#^Linear regression analysis was performed and p-values indicate whether slope is significantly different from zero (α = 0.05).

*Quantification of total protein (µBCA) for release was performed 6 months after production. Therefore, stability testing after 3 and 6 months was not performed.

**Test was implemented as part of the surveillance test and stability panel about 6 months after manufacture and was therefore applied for the first time at the 9 months stability time point.

^&^Surveillance analyses at the end of stability were performed at GVGH only at 36 months. Stability panel analyses were shifted to 42 months and were conducted at Northway Biotech (Vilnius, Lithuania).

**Supplementary Table 3**

**(A) Results of accelerated stability testing of STmGMMA/Alhydrogel toxicology lot R-0007-02 at 25°C**

|  |  | **Analysis outcome (RSD%) at sampling time in days** | | | | | | | | |  | |
| --- | --- | --- | --- | --- | --- | --- | --- | --- | --- | --- | --- | --- |
| **Test** | **Acceptance criteria** | **0** | **1** | **2** | **4** | **7** | **14** | **28** | **56** | **Slope^§^  (p value)** | |  |
| Quantification of OAg | Report µg OAg/mL | 115.0  (7.6) | nd | 117.0  (3.0) | nd | 112.6  (9.1) | 120.2  (11.9) | 136.5  (1.7) | 126.5  (7.8) | ns  (0.144) | |  |
| Quantification of protein not adsorbed to aluminium hydroxide | <10% | <10% | <10% | <10% | <10% | <10% | <10% | <10% | <10% | NA | |  |
| pH | 6.0 – 7.0 | 6.58 | 6.53 | 6.52 | 6.50 | 6.49 | 6.64 | 6.54 | 6.53 | ns  (0.960) | |  |
| Osmolality | 240 - 360 mOsm/kg | 320.0  (0.6) | 321.3  (0.4) | 320.7  (0.2) | 319.7  (0.5) | 319.7  (0.2) | 321.7  (0.5) | 324.3  (0.4) | 321.3  (0.2) | ns  (0.248) | |  |
| Particle Size  [D4,3] | <10 µm | 10.9  (4.7) | 7.8  (2.2) | 7.6  (1.2) | 8.9  (2.0) | 9.9  (6.6) | 10.3  (2.2) | 8.6  (2.0) | 8.0  (4.7) | ns  (0.514) | |  |
| Particle size  [D90] | For information | 20.8  (1.1) | 13.2 (3.0) | 13.0  (1.1) | 15.6  (1.9) | 20.3  (11.2) | 26.3  (2.2) | 21.8  (2.5) | 14.5 (12.8) | ns  (0.992) | |  |

ns: not significant

s: significant

§Slope over time different from zero, assessment of significance comparing calculated t-statistic with Student t-value for the data set (based on probability 0.05, one-tailed distribution, degrees of freedom)

Time point 1 and 4 days is not part of the stability testing by FAcE

**(B) Results of accelerated stability testing of STmGMMA/Alhydrogel toxicology lot R-0007-02 at 37°C**

|  |  | **Analysis outcome (RSD%) at sampling time in days** | | | | | | | |  |
| --- | --- | --- | --- | --- | --- | --- | --- | --- | --- | --- |
| **Test** | **Acceptance criteria** | **0** | **1** | **2** | **4** | **7** | **14** | **28** | **56** | **Slope^§^  (p value)** |
| Quantification of OAg | Report µg OAg/mL | 115.0  (7.6) | Nd* | 113.2  (4.8) | Nd* | 119.9  (10.0) | 147.0  (5.5) | 142.5  (7.1) | 89.9  (9.4) | ns  (0.465) |
| Quantification of protein not adsorbed to aluminium hydroxide | <10% | <10% | <10% | <10% | <10% | <10% | <10% | <10% | <10% | NA |
| pH | 6.0 – 7.0 | 6.58 | 6.51 | 6.52 | 6.52 | 6.58 | 6.67 | 6.55 | 6.58 | ns  (0.539) |
| Osmolality | 240 - 360 mOsm/kg | 320.0  (0.6) | 320.3  (0.5) | 319.0  (0.3) | 319.3  (0.2) | 321.0  (0.5) | 319.0  (0.3) | 321.7  (0.2) | 320.7  (0.2) | ns  (0.253) |
| Particle Size  [D4,3] | <10 µm | 10.9  (4.7) | 8.1  (2.9) | 8.2  (5.3) | 8.1  (1.6) | 11.5  (17.3) | 10.6  (3.3) | 9.5  (3.6) | 8.2  (26.0) | ns  (0.610) |
| Particle size  [D90] | For information | 20.8  (1.1) | 14.4  (3.0) | 14.0  (3.7) | 13.5  (1.7) | 24.4  (3.7) | 26.8  (4.0) | 25.6  (5.0) | 14.1  (26.0) | ns  (0.996) |

nd: time points not done due to conflict with duration of assay

ns: not significant

s: significant

§Slope over time different from zero, assessment of significance comparing calculated t-statistic with Student t-value for the data set (based on probability 0.05, one-tailed distribution, degrees of freedom)

*Time point 1 and 4 days is not part of the stability testing by FAcE

**(C) Results of accelerated stability testing of SEnGMMA/Alhydrogel toxicology lot R-0007-01 at 25°C**

|  |  | **Analysis outcome (RSD%) at sampling time in days** | | | | | | | |  |
| --- | --- | --- | --- | --- | --- | --- | --- | --- | --- | --- |
| **Test** | **Acceptance criteria** | **0** | **1** | **2** | **4** | **7** | **14** | **28** | **56** | **Slope^§^**  **(p value)** |
| Quantification of OAg | Report µg OAg/mL | 72.5  (18.2) | nd* | 70.1  (26.4) | nd* | 78.6  (4.0) | 62.0  (12.1) | 75.2  (9.8) | 65.6  (5.0) | ns  (0.511) |
| Quantification of protein not adsorbed to aluminium hydroxide | <10% | <10% | <10% | <10% | <10% | <10% | <10% | <10% | <10% | NA |
| pH | 6.0 – 7.0 | 6.57 | 6.53 | 6.53 | 6.50 | 6.51 | 6.65 | 6.53 | 6.56 | ns  (0.674) |
| Osmolality | 240 - 360 mOsm/kg | 319.3 | 319.3 | 318.7 | 318.3 | 318.3 | 318.7 | 321.3 | 319.3 | ns  (0.328) |
| Particle Size  [D4,3] | <10 µm | 8.6  (4.3) | 7.1  (1.3) | 8.6  (2.1) | 8.4  (3.6) | 9.5  (2.5) | 10.6  (3.3) | 11.9  (10.1) | 9.6  (16.0) | ns  (0.191) |
| Particle size  [D90] | For information | 16.4  (4.2) | 13.0  (2.7) | 17.3  (3.0) | 13.7  (2.5) | 21.0  (4.5) | 26.8  (4.0) | 30.4  (4.0) | 26.5  (16.5) | s  (0.041) |

conf.: conform

nd: time points not done due to conflict with duration of assay

ns: not significant

s: significant

§Slope over time different from zero, assessment of significance comparing calculated t-statistic with Student t-value for the data set (based on probability 0.05, one-tailed distribution, degrees of freedom)

*Time points 1 and 4 days are not part of the stability testing by FAcE.

**(D) Results of accelerated stability testing of SEnGMMA/Alhydrogel toxicology lot R-0007-01 at 37°C**

|  |  | **Analysis outcome (RSD%) at sampling time in days** | | | | | | | |  |
| --- | --- | --- | --- | --- | --- | --- | --- | --- | --- | --- |
| **Test** | **Acceptance criteria** | **0** | **1** | **2** | **4** | **7** | **14** | **28** | **56** | **Slope^§^  (p value)** |
| Quantification of OAg | Report µg OAg/mL | 72.5  (18.2) | nd | 82.8  (9.0) | nd | 84.9  (18.3) | 90.0  (6.7) | 79.6  (3.4) | 77.7  (12.8) | ns  (0.772) |
| Quantification of protein not adsorbed to aluminium hydroxide | <10% | <10% | <10% | <10% | <10% | <10% | <10% | <10% | <10% | NA |
| pH | 6.0 – 7.0 | 6.57 | 6.54 | 6.54 | 6.53 | 6.55 | 6.64 | 6.57 | 6.58 | ns  (0.381) |
| Osmolality | 240 - 360 mOsm/kg | 319.3  (0.4) | 318.0  (0.6) | 318.3  (0.2) | 319.7  (0.5) | 319.7  (0.4) | 312.3  (0.2) | 318.7  (0.4) | 319.0(0.5) | ns  (0.994) |
| Particle Size  [D4,3] | <10 µm | 8.6  (4.3) | 9.7  (2.0) | 9.2  (2.7) | 7.7  (3.5) | 22.4  (15.1) | 11.0  (4.1) | 16.8  (4.7) | 7.2  (3.4) | ns  (0.860) |
| Particle size  [D90] | For information | 16.4  (4.2) | 27.0  (2.8) | 24.6  (3.1) | 13.9  (5.3) | 37.8  (7.6) | 32.3  (4.2) | 50.9  (4.3) | 19.9  (3.7) | ns  (0.701) |

NA: not applicable

nd: time points not done due to conflict with duration of assay

ns: not significant

s: significant

§Slope over time different from zero, assessment of significance comparing calculated t-statistic with Student t-value for the data set (based on probability 0.05, one-tailed distribution, degrees of freedom)

Time points 1 and 4 days are not part of the stability testing by FAcE
